# Oxime radicals: generation, properties and application in organic synthesis

**DOI:** 10.3762/bjoc.16.107

**Published:** 2020-06-05

**Authors:** Igor B Krylov, Stanislav A Paveliev, Alexander S Budnikov, Alexander O Terent’ev

**Affiliations:** 1 N. D. Zelinsky Institute of Organic Chemistry, Russian Academy of Sciences, Leninsky Prospect 47, Moscow, 119991, Russia

**Keywords:** iminoxyl radicals, isoxazolines, oxidative cyclization, oxime radicals, oximes

## Abstract

*N*-Oxyl radicals (compounds with an N–O^•^ fragment) represent one of the richest families of stable and persistent organic radicals with applications ranging from catalysis of selective oxidation processes and mechanistic studies to production of polymers, energy storage, magnetic materials design and spectroscopic studies of biological objects. Compared to other *N*-oxyl radicals, oxime radicals (or iminoxyl radicals) have been underestimated for a long time as useful intermediates for organic synthesis, despite the fact that their precursors, oximes, are extremely widespread and easily available organic compounds. Furthermore, oxime radicals are structurally exceptional. In these radicals, the N–O^•^ fragment is connected to an organic moiety by a double bond, whereas all other classes of *N*-oxyl radicals contain an R_2_N–O^•^ fragment with two single C–N bonds. Although oxime radicals have been known since 1964, their broad synthetic potential was not recognized until the last decade, when numerous selective reactions of oxidative cyclization, functionalization, and coupling mediated by iminoxyl radicals were discovered. This review is focused on the synthetic methods based on iminoxyl radicals developed in the last ten years and also contains some selected data on previous works regarding generation, structure, stability, and spectral properties of these *N*-oxyl radicals. The reactions of oxime radicals are classified into intermolecular (oxidation by oxime radicals, oxidative C–O coupling) and intramolecular. The majority of works are devoted to intramolecular reactions of oxime radicals. These reactions are classified into cyclizations involving C–H bond cleavage and cyclizations involving a double C=C bond cleavage.

## Introduction

**Figure 1:**
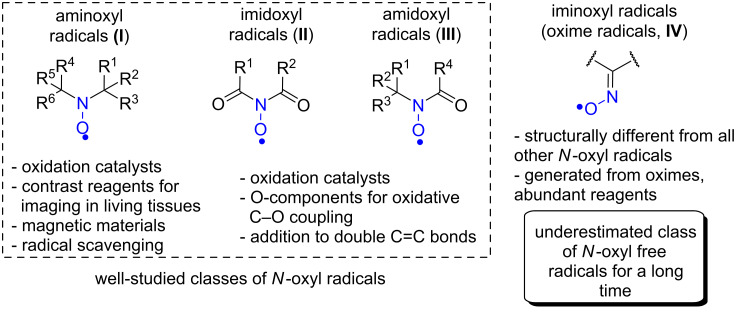
Imine-N-oxyl radicals (**IV**) discussed in the present review and other classes of N-oxyl radicals (**I**–**III**).

Free radicals in which an unpaired electron is localized on the N–O fragment (*N*-oxyl radicals, Figure 1) occupy a special place in organic chemistry due to the increased stability and ease of generation, the diversity of their structures, properties, and applications.

Stable *N*-oxyl radicals (mainly of the aminoxyl type, Figure 1, **I**) are used in the development of organic magnetic materials [1], organic batteries [2–4], in the preparation of polymers by living polymerization [5–6], in the studies of biomolecules and living systems by EPR [7] and NMR [8] techniques. Stable *N*-oxyl radicals occupy a central place in organic chemistry as scavengers of C-centered radicals [9] and selective oxidation organocatalysts (for example, in the oxidation of alcohols to the corresponding carbonyl compounds [10–11]). Recently, highly reactive imidoxyl radicals (Figure 1, **II**) have found a wide application in the processes of hydrogen atom abstraction with cleavage of the C–H bond [12–18] and in the processes of functionalization of С=С double bonds [19–20]. Amidoxyl radicals (Figure 1, **III**) are applied in the functionalization of the double bonds [21–26] and in mild oxidations [27].

In contrast to the mentioned *N*-oxyl radical classes (Figure 1, **I**–**III**) which have two single nitrogen–carbon bonds, the iminoxyl radicals (also known as oxime radicals, Figure 1, **IV**) have a carbon–nitrogen double bond. This structural feature is responsible for principal differences in the electronic structure, spectral properties, and chemical reactivity between oxime radicals and other types of *N*-oxyl radicals.

For a long time, the synthetic potential of iminoxyl radicals remained underestimated and their chemistry was mainly represented by fundamental physico-chemical studies. The precursors of imine-*N*-oxyl radicals are oximes, a widely available and fundamental class of organic compounds. However, oxime radicals almost did not find synthetic use until recently, probably due to the low stability of the majority of representatives of this type of radicals. The applications of oxime radicals in organic synthesis have developed rapidly during the last years, and we believe that this review is essential for the demonstration of a new face of the chemistry of this class of *N*-oxyl radicals. There are reports on the iminoxyl radical involvement in redox processes occurring in living organisms, for example, in microsomal oxidation of *N*-hydroxyguanidines and amidoximes [28], oxidation of tyrosine phenolic moiety in the presence of NO [29–33].

This review focuses on the synthetic use of oxime radicals. Most of the works on this topic have been published over the past ten years. In most cases, these are intramolecular reactions of oxidative cyclization. Examples of intermolecular reactions of oxime radicals, a brief description of their structure, stability, and spectral properties are also given. The chemistry of iminoxyl radicals (including their generation, structure, EPR spectroscopy, and reactions) was reviewed in 1978 before the discovery of their substantial synthetic potential [34]. A book chapter was dedicated to the chemistry of stable di-*tert*-alkyl iminoxyl radicals [35]. Kinetic [36] and EPR data [37] of iminoxyl radicals were previously compiled in tabular form.

## Review

### General information about iminoxyl radicals: generation, structure, stability, and spectral data

Iminoxyl radicals were first discovered in 1964 by EPR spectroscopy as short-living intermediates formed from the oximes of both aromatic and aliphatic ketones and aldehydes, as well as from the oximes of quinones under the action of a strong single-electron oxidant, cerium(IV) ammonium nitrate, in methanol [38]. To record EPR spectra, a flow system was used, which allowed observation of radicals with lifetimes of about 10^−2^ s [39]. The EPR spectra of iminoxyl radicals are characterized by large values of the hyperfine splitting constants of an unpaired electron with a ^14^N nucleus (a^N^ ≈ 28–33 G [35–38]), which are very different from those for other *N*-oxyl radicals, such as imidoxyl (a^N^ ≈ 4.2–4.9 G [40]), amidoxyl (a^N^ ≈ 5–8 G [41–42]), and aminoxyl (a^N^ ≈ 15 G [43]). The characteristic ^14^N hyperfine splitting constant makes EPR spectroscopy a convenient method for the identification of iminoxyl radicals, and for many of them, EPR is the only observation method due to low stability, and therefore low concentration in investigated systems. For the most stable iminoxyl radicals, sufficiently concentrated solutions were obtained and investigated by IR spectroscopy. The IR spectra show the absence of the line of stretching vibrations of the O–H bond, characteristic of the parent oximes, as well as the appearance of a new intense band corresponding to the asymmetric vibrations of the C=N–O^•^ fragment (1550 сm^−1^ for the diacetyliminoxyl radical [44], 1595 сm^−1^ for the di(1-adamantyl)iminoxyl, and 1610 сm^−1^ for the di-*tert*-butyliminoxyl radical [45]).

Various oxidants were used for the generation of iminoxyl radicals from oximes, including transition metal compounds, such as (NH_4_)_2_Ce(NO_3_)_6_ [38,46], Fe(ClO_4_)_3_ [44,46], Cu(ClO_4_)_2_ [46], Pb(OAc)_4_ [44,46–51], PbO_2_ [52], Mn(OAc)_3_ [46], KMnO_4_ [46], Ag_2_O [53], AgO [54], Horseradish peroxidase/H_2_O_2_ [55], metal-free oxidants PhI(OAc)_2_ [46], *t*-BuOO*t*-Bu [53] or quinones [56] under UV irradiation. Anodic oxidation was also reported [57].

**Figure 2:**
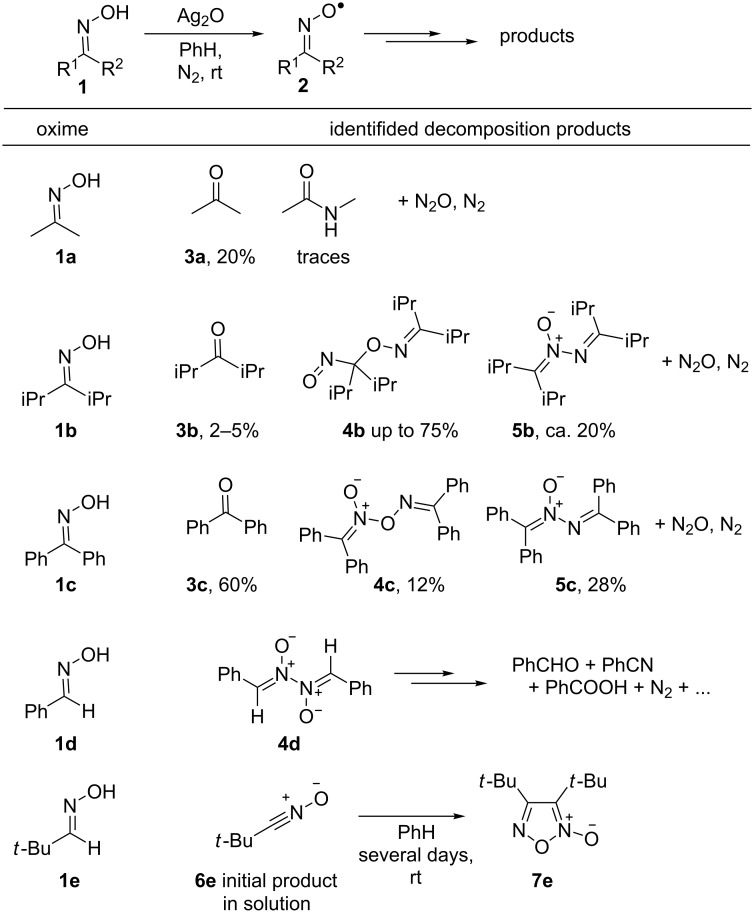
The products of decomposition of iminoxyl radicals generated from oximes by oxidation with Ag_2_O.

The establishing of the self-decay pathways of iminoxyl radicals is complicated by the formation of a large number of products, some of the initially formed products are not stable. Moreover, participation of the oxidizing agent not only in the radical generation, but also in its decay also possible [53]. The products formed during the decomposition of iminoxyl radicals **2** generated from oximes **1** under the action of Ag_2_O [53] were studied by K. U. Ingold et al. (Figure 2).

In most cases, the reaction was accompanied by the release of N_2_ and N_2_O, as well as the corresponding carbonyl compounds (**3a**–**c**). Dimerization of iminoxyl radicals with the formation of a C–O bond (dimer **4b**, oxidation of diisopropyl oxime **1b**), an O–N bond (dimer **4c,** oxidation of benzophenone oxime **1c**), and an N–N bond (dimer **4d**, oxidation of benzaldoxime **1d**, see also [58]) was observed. As a rule, the initial dimers of iminoxyl radicals are unstable, which makes their analysis difficult. Azine-*N*-oxides **5b**,**c** were also obtained in significant amounts from oximes **1b**,**c** (yields 20–28%). The C–O dimeric product **4b** of the diisopropyl iminoxyl radical is unstable at room temperature even in solution. At the same time, it gives a sufficiently strong EPR signal corresponding to the free iminoxyl radical, which indicates the reversibility of dimerization [53]. During the oxidation of pivalic aldoxime **1e** by Ag_2_O, the formation of nitrile oxide **6e** was observed, which then slowly dimerized to the corresponding furoxan **7e**.

**Scheme 1:**
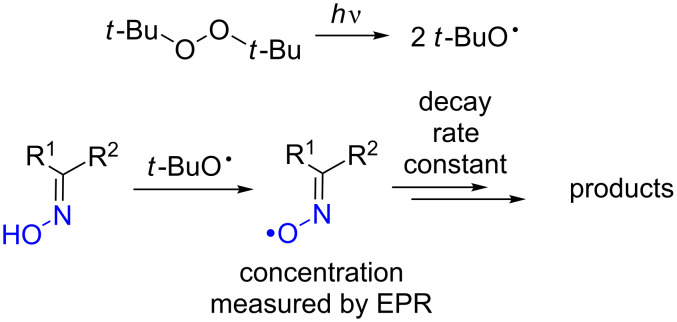
Generation of oxime radicals and study of the kinetics of their decay by photolysis of the solution of *t*-BuOO*t*-Bu and oxime in an EPR spectrometer cavity.

The kinetics of the decomposition of dialkyl, arylalkyl, and diaryl oxime radicals was also studied by EPR spectroscopy [53]. Radicals were generated under inert atmosphere directly in the EPR cavity by photolysis of the added di-*tert*-butyl peroxide (Scheme 1).

The authors pointed out the complexity of the processes of iminoxyl radicals’ decomposition and the difficulties associated with the interpretation of the obtained data [53]. During EPR monitoring of the generation and decomposition of iminoxyl radicals, the formation of several free-radical products of a non-iminoxyl type, probably of the general formula R^1^R^2^NO^•^, was observed. It was established that the studied iminoxyl radicals reversibly dimerized in the solution. For sterically unhindered dialkyliminoxyl radicals, the radical–dimer equilibrium was quickly reached, shifted toward the dimer, while a first-order decay kinetics of was observed for the iminoxyl radical. For sterically hindered *tert*-butylmethyliminoxyl and diisopropyliminoxyl radicals, as well as for diaryl and alkylaryliminoxyl radicals, the radical–dimer equilibrium was reached slowly, it was shifted toward the free radical, and a second-order decay kinetics was observed.

**Scheme 2:**
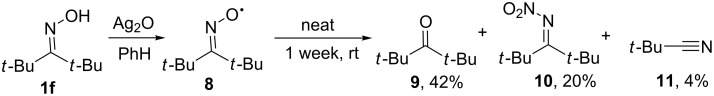
Synthesis of di-*tert*-butyliminoxyl radical and its decomposition products.

The first synthesized long-lived iminoxyl radical that did not undergo decomposition and dimerization in the solution for a time sufficient to use it as a reagent was the di-*tert*-butyliminoxyl radical (**8**) [45,59]. It was obtained by oxidation of di-*tert*-butyl ketoxime (**1f**) with silver(I) oxide (Ag_2_O) in benzene at 25 °C (Scheme 2). This radical is stable at 25 °C in *n*-hexane. In pure form it is storable only at −78 °C as a solid. At room temperature, radical **8** is a blue oil. When storing **8** in the dark without solvent at 25 °C for a week, the following decomposition products were identified: di-*tert*-butyl ketone (**9**, 42%), di-*tert*-butyl nitroimine (**10**, 20%), and pivalonitrile (**11**, 4%) [45].

**Scheme 3:**
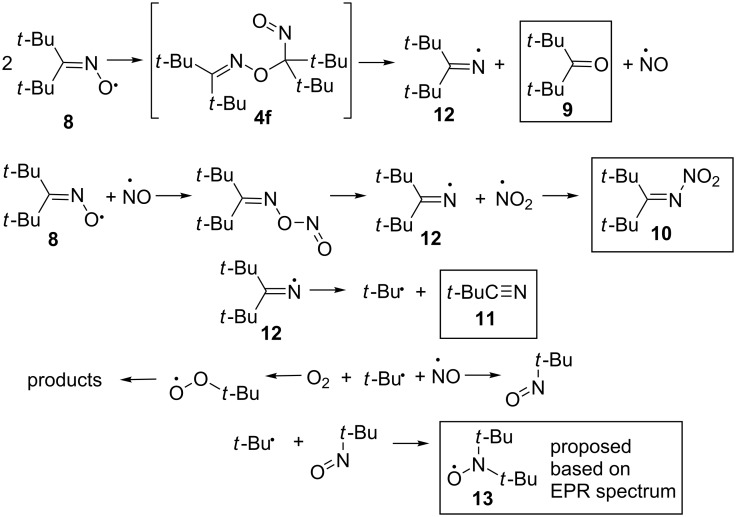
The proposed reaction pathway of the decomposition of di-*tert*-butyliminoxyl radical (experimentally identified products are highlighted by rectangles).

The proposed scheme for the decomposition of di-*tert*-butyliminoxyl radical (**8**) is presented in Scheme 3 [35,45]. It includes formation of C–O dimer **4f** followed by the fragmentation to iminyl radical **12**, ketone **9**, and nitric oxide. The formation of nitroimine **10** is explained by the interaction of oxime radical **8** with nitric oxide. Pivalonitrile (**11**) is presumably formed via β-scission of iminyl radical **12** (Scheme 3). During the decomposition of oxime radical **8** an EPR signal typical for a dialkyl aminoxyl radical (type **I** in Figure 1) was also observed, it was assigned to the di-*tert*-butyl nitroxyl radical (**13**).

The proposed pathway of *N*-nitroimine **10** formation (Scheme 3) was confirmed by additional experiments. It was established that the di-*tert*-butyliminoxyl radical was not stable in NO atmosphere, the reaction proceeds at room temperature for an hour, and *N*-nitroimine **10** is formed [45].

**Scheme 4:**
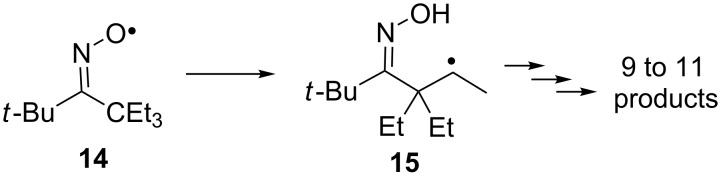
Monomolecular decomposition of the *tert*-butyl(triethylmethyl)oxime radical.

Attempts to increase the stability of the iminoxyl radical by replacing the *tert*-butyl substituent with a bulkier triethylmethyl or other acyclic *tert*-alkyl substituents were not successful. In the case of Me_3_C(Et_3_C)C=NO^•^ radical **14**, a monomolecular decomposition process was proposed, associated with an intramolecular hydrogen atom abstraction by an iminoxyl radical leading to the intermediate **15** (Scheme 4) [35,60–61].

**Scheme 5:**
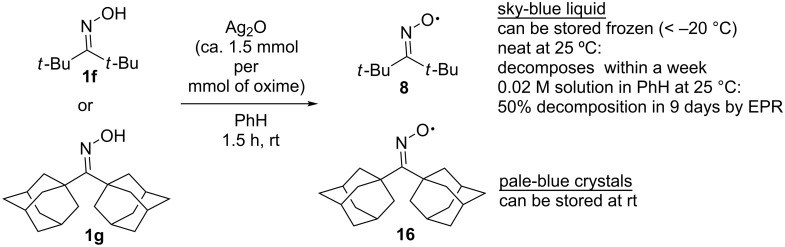
The synthesis and stability of the most stable dialkyl oxime radicals – di-*tert*-butyliminoxyl and di-(1-adamantyl)iminoxyl.

In 1974, a di(1-adamantyl)iminoxyl radical **16** was synthesized analogously to di-*tert*-butyliminoxyl radical (**8**) from the corresponding oxime **1g** (Scheme 5). Oxime radical **16** is a pale blue crystalline compound that is stable at room temperature [35,62], whereas liquid neat di-*tert*-butyliminoxyl radical (**8**) decomposed within a week [59]. The di(1-adamantyl)iminoxyl radical (**16**) was characterized by IR, UV–vis, EPR, and NMR spectroscopy, and its dipole moment in the benzene solution was measured (2.90 D).

**Scheme 6:**
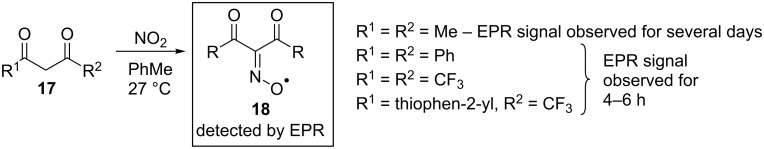
The formation of iminoxyl radicals from β-diketones under the action of NO_2_.

Besides the mentioned sterically hindered iminoxyl radicals **8** and **16**, iminoxyl radicals with electron-withdrawing substituents at the C=NO^•^ fragment also demonstrate increased stability compared to ordinary alkyl and aryl iminoxyl radicals. For example, a number of long-lived diacyl iminoxyl radicals **18** were generated by the action of tetranitromethane [63] or NO_2_ [64] on the corresponding β-diketones **17** or barbituric acid. The formation and decay of radicals were studied by EPR spectroscopy [64]. The lifetimes of radicals in the solution ranged from several hours to several days (Scheme 6) [63–64].

**Scheme 7:**
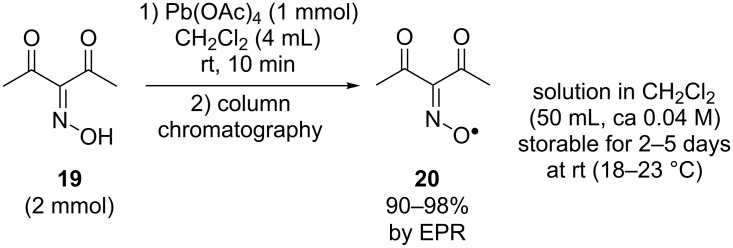
Synthesis of the diacetyliminoxyl radical.

Recently, a method for the preparative synthesis of diacetyliminoxyl radical **20** in high yield via the oxidation of diacetyl oxime **19** by Pb(OAc)_4_ was developed [44] (Scheme 7). The resulting radical can be stored for 2–5 days in the solution at room temperature without decomposition according to EPR and IR spectroscopy. Compound **20** represents a very rare example of sterically unhindered, but nonetheless extremely persistent oxime radical.

**Scheme 8:**
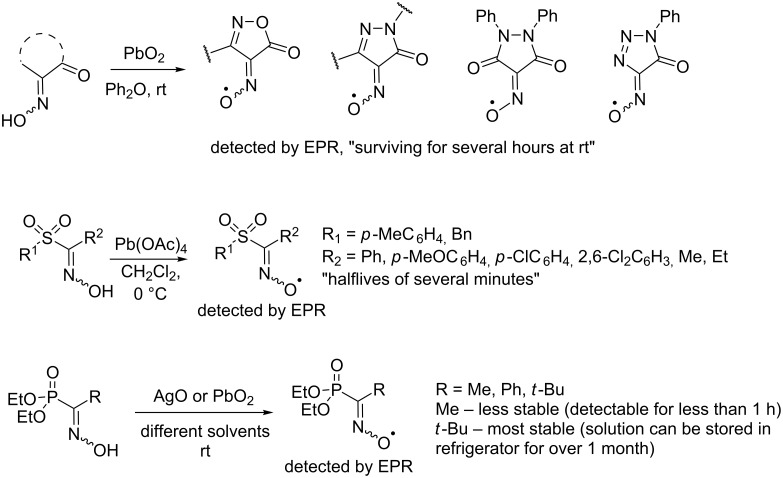
Examples of long-living oxime radicals with electron-withdrawing groups and the conditions for their generation.

Other long-living oxime radicals with electron-withdrawing substituents are also known, for example, based on N-containing heterocycles (isoxazolones, pyrazolones, pyrazolidin-3,5-diones, and 1,2,3-triazolones [52]), sulfones [65], and phosphonates [54] (Scheme 8).

Based on the data of EPR spectroscopy [35,38,49–50,66] and quantum chemical calculations [67], the maximum spin density in iminoxyl radicals is located on oxygen and on nitrogen. A lone electron pair of sp^2^ hybridized nitrogen located in the plane of the C=N–O fragment serves for the delocalization of an unpaired electron. Thus the unpaired electron is localized on the orbital that is orthogonal to the C=N π-bond, and therefore, the oxime radicals are considered as σ-radicals [35]. The electronic structure of the iminoxyl radical can be represented by two main resonance forms presented below. The calculated and experimental data indicate that the localization of an unpaired electron on the NO fragment is also valid for the case of arylalkyl and diaryl oximes – conjugation of the radical center with π-systems of aryl rings is not observed [47,49–50,67].

**Figure 3:**
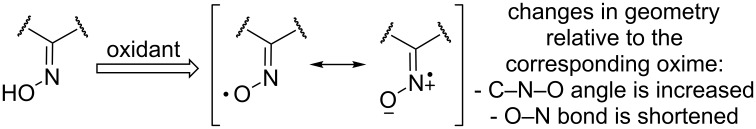
The electronic structure iminoxyl radicals and their geometry compared to the corresponding oximes.

It is known that the angular structure is characteristic for the CNO fragment of oxime radicals, and in the case of different substituents at the carbon atom, two isomers (*E* and *Z*) exist. The isomerization of oxime radicals proceeds much easier than for the corresponding oximes; the observation of individual isomers is generally possible only at low temperatures [68–69] (about 190 K). According to quantum chemical calculations, the oxime radicals have an increased C=N–O angle and a shortened N–O bond compared to the corresponding oximes (Figure 3) [44,52,70].

**Figure 4:**
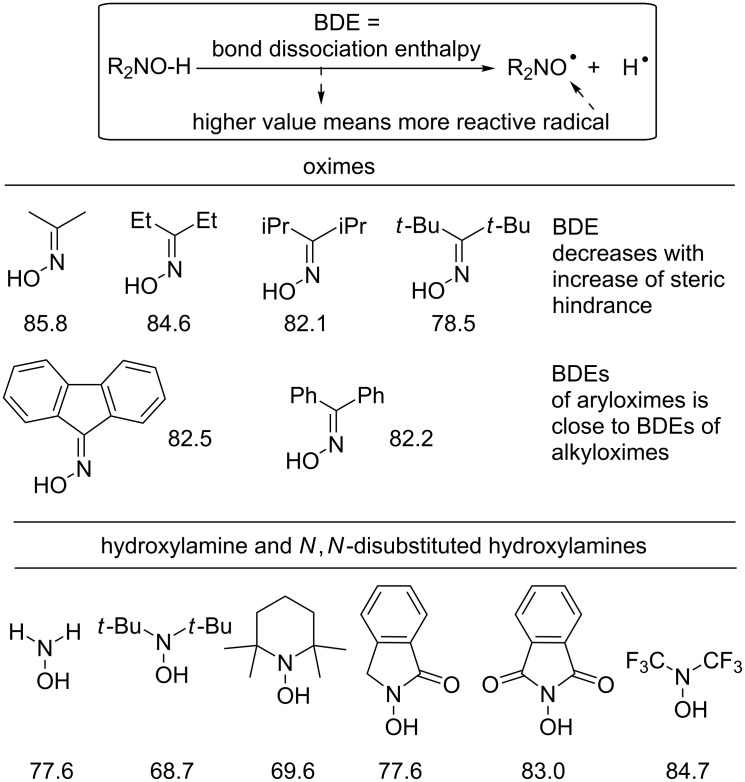
Bond dissociation enthalpies (kcal/mol) of oximes and *N*,*N*-disubstituted hydroxylamines calculated on UB3LYP/6-311+G(d,p) level using an isodesmic reaction referencing the experiment BDE of phenol.

One of the important quantitative values that determine the reactivity of O radicals is the O–H bond dissociation enthalpy (BDE) in the parent OH compound (Figure 4). This value affects both the ease of the generation of radicals from the corresponding OH compounds and the oxidative properties of the O radicals. The O–H BDE values were determined for a number of oximes by the computational [67,70–71] and experimental [70,72] methods. It was established that the BDE decreased with an increase in the volume of substituents at the C=NOH fragment, which was consistent with the spatial structure of the oxime radicals – an increase in the C=N–O angle in the radical compared to the oxime led to a decrease in steric repulsion between the substituents at the carbon atom and the oxygen atom. It should also be noted that there is no noticeable decrease in the O–H BDE in diaryl oximes compared to dialkyl oximes (some examples are shown in Figure 4 [71]), which is consistent with the idea that an unpaired electron is delocalized by the conjugation with a lone pair of the nitrogen atom, but not by the conjugation with the π-system of the molecule.

Figure 4 also shows examples of the calculated values of the O–H BDE of non-oxime compounds with a NOH fragment [71]. The O–H bond in oximes is stronger than in hydroxylamines with a similar structure, except for hydroxylamines with strong electron-withdrawing groups (such as carbonyl and CF_3_).

Оxime radicals did not find wide application in organic synthesis and were mainly the subject of fundamental physicochemical studies for a long time since their discovery in 1964. The possible reason is the low stability of the majority of iminoxyl radicals. Only the relatively stable di-*tert*-butyliminoxyl radical was studied as a reagent in oxidative transformations of various substrates, such as unsaturated hydrocarbons, phenols, amines, and organometallic compounds. A breakthrough in the synthetic use of iminoxyl radicals has occurred in recent years when they found a wide application in intramolecular processes of oxidative cyclization with functionalization of C–H and С=С fragments.

In the majority of works related to synthetic use of oxime radicals, intramolecular reactions are reported. Perhaps this is due to the low stability of oxime radicals. The main preparative reactions involving oxime radicals include the addition of the oxime radical to the C=C double bond or hydrogen atom abstraction. Due to the delocalization of the unpaired electron between the oxygen and nitrogen atoms in the oxime radicals, they can form both C–O and C–N bonds. As a rule, a C–O bond is formed in intermolecular reactions, intramolecular cyclization generally occurs with the formation of a five-membered cycle of isoxazoline (C–O bond formation) or nitrone (C–N bond formation).

### Application of the oxime radicals in organic synthesis: intermolecular reactions

Selective intermolecular reactions involving oxime radicals are relatively rare compared with intramolecular ones. Many of these reactions involve a stable di-*tert*-butyliminoxyl radical. Violuric acid and *N*,*N*’-dimethylvioluric acid, precursors of the corresponding persistent iminoxyl radicals, were studied as mediators for the electrochemical oxidation of lignin [73] and enzymatic oxidations [74–76] but have not been widely used in organic synthesis.

**Scheme 9:**
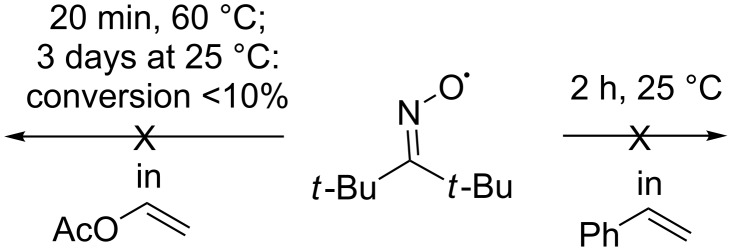
Examples demonstrating the low reactivity of the di-*tert*-butyliminoxyl radical towards the substrates with double С=С bonds – styrene and vinyl acetate.

The di-*tert*-butyliminoxyl radical proved to be quite unreactive with respect to a C=C double bond containing substrates that are considered as effective scavengers of free radicals (Scheme 9). It remains unchanged [45] when dissolved in styrene (2 hours, 25 °C) or vinyl acetate (20 minutes, 60 °C; conversion of the oxime radical was less than 10% after 3 days at 25 °C). The inertness of the di-*tert*-butyliminoxyl radical with respect to the mentioned substrates with a C=C double bond was explained by the steric hindrance of the iminoxyl radical.

**Scheme 10:**
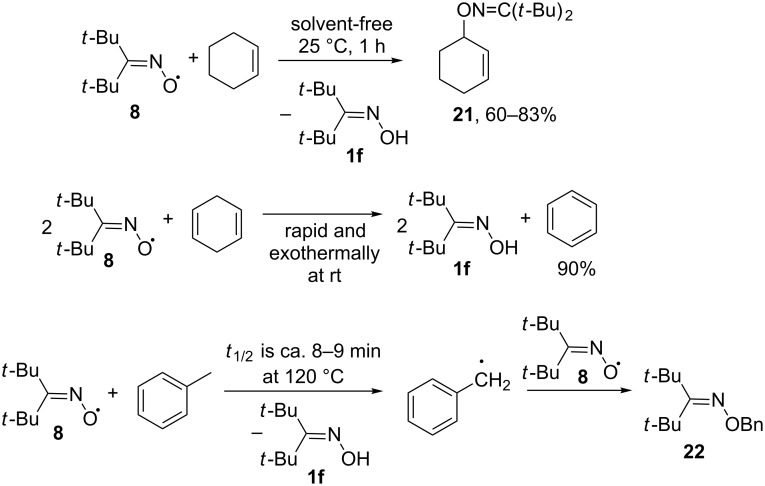
The reactions of di-*tert*-butyliminoxyl radical with unsaturated hydrocarbons involving hydrogen atom abstraction.

On the other hand, di-*tert*-butyliminoxyl radical (**8**) can react with unsaturated hydrocarbons by abstracting the hydrogen atom from the allyl or benzyl position (Scheme 10) [35,45,60–61]. The C-centered radicals formed after hydrogen atom abstraction from the allyl or benzyl position couple with the di-*tert*-butyliminoxyl radical forming the oxime ethers **21** and **22**. 1,4-Cyclohexadiene is dehydrogenated to benzene instantly and exothermally at room temperature [45]. The solvent-free reaction of oxime radical **8** and cyclohexene takes 1 h at 25 °C [61]. The benzyl hydrogen atoms are abstracted at higher temperatures [35,45].

**Scheme 11:**
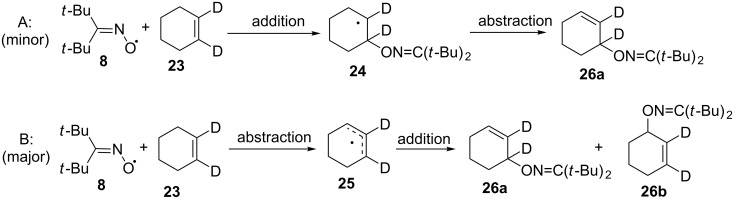
Possible mechanisms of reaction of di-*tert*-butyliminoxyl radical with alkenes.

Reactions of di-*tert*-butyliminoxyl radical (**8**) with allylic moieties can theoretically occur by two pathways that give the same final product – the *O*-allyl derivative of the oxime [35,77]. In the first pathway (Scheme 11A), the initial addition of the di-*tert*-butyliminoxyl radical to the double bond is followed by hydrogen atom abstraction from the resulting alkyl radical **24**. The second pathway (Scheme 11B) begins with the abstraction of an allylic hydrogen atom, then the di-*tert*-butyliminoxyl radical adds to either end of the resulting allylic radical **25**. However, the results of pathways A and B are different for selectively deuterated cyclohexene **23** in Scheme 11. The addition–abstraction pathway (A) results in a single deuterated product **26a**, whereas the abstraction–addition pathway (B) gives two products: the product obtained by pathway A (**26a**) and a product **26b** unique to pathway B. The abstraction–addition process (B) is dominant for three alkenes studied, namely, cyclohexene, cyclooctene, and 3-hexene, with 90–92% of the overall reaction occurring by this mechanism [77].

The reactions of the di-*tert*-butyliminoxyl radical with phenol and its derivatives are faster than with alkenes. The highest reaction rates are observed in the case of electron-donating substituents (Y) in 4-YC_6_H_4_OH [35,78]. The hydrogen atom abstraction rate accelerating effect of electron-donating substituents was explained by the decrease of the O–H bond dissociation energy by the electron-donating substituent Y [35,79–84].

**Scheme 12:**
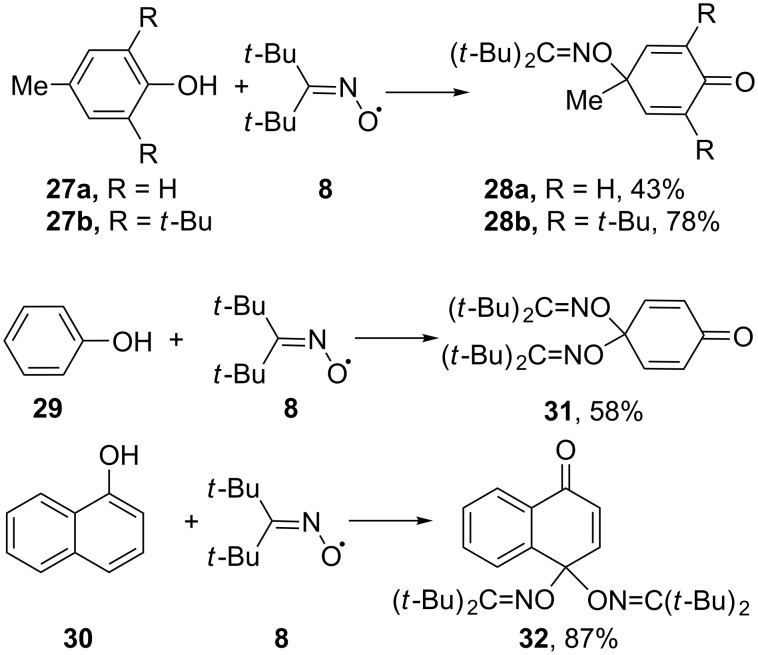
Products of the reaction between di-*tert*-butyliminoxyl radical and phenol derivatives.

The result of these reactions depends on the phenol structure. 4-Methylphenol (**27a**) and 2,6-di-*tert*-butyl-4-methylphenol (BHT, **27b**) gave 4-methyl-4-iminooxycyclohexadienones **28a,b** (Scheme 12). Phenol (**29**) and 1-naphthol (**30**) were transformed into 4,4-bisoximes **31** and **32**, respectively (Scheme 12) [35,78].

**Scheme 13:**
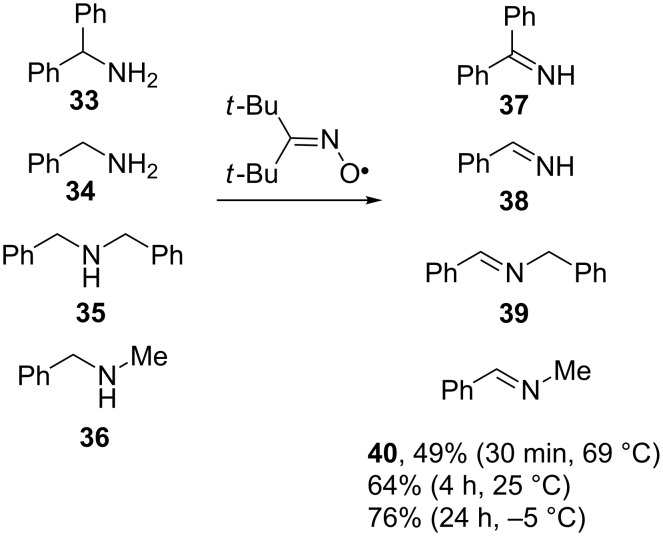
The reaction of di-*tert*-butyliminoxyl radical with amines.

Imines **37–40** were obtained with good yields by the reaction of di-*tert*-butyliminoxyl radicals with primary and secondary amines **33–36** for several hours at room temperature in pentane or hexane (Scheme 13) [85]. Due to the low stability of most imines, they were not isolated in pure form but were transformed into 2,4-dinitrophenylhydrazones. For example, the yields of 2,4-dinitrophenylhydrazones from **37**, **38** and **39** were 79, 68 and 78%, respectively [85]. The example of *N*-benzylidenemethylamine (**40**), shows the dependence of the imine yield on reaction time and temperature (Scheme 13). Higher yields of **40** were achieved at lower temperatures but longer reaction times were necessary in this case [85].

**Scheme 14:**
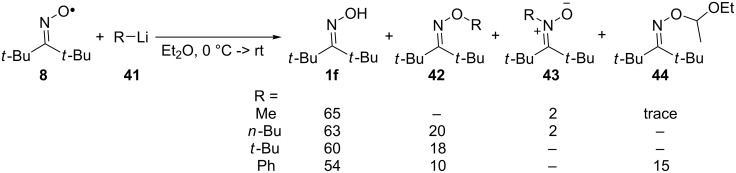
Reaction of di-*tert*-butyliminoxyl radicals with organolithium reagents.

Di-*tert*-butyliminoxyl radicals react with Grignard reagents and organolithium reagents **41** at 0 °C in Et_2_O forming mainly di-*tert*-butyl oxime **1f** and the products of C–O coupling [86]. The reactions were performed by addition of the solution of the organometallic compound **41** in Et_2_O to the solution of di-*tert*-butyliminoxyl radical in Et_2_O. The product yields obtained employing organolithium reagents are presented below (Scheme 14). The Grignard reagents demonstrated very similar results that are omitted.

Among the major C–O coupling product (oxime ether **42**) small amounts of C–N coupling products (nitrones **43**) were detected in the case of sterically unhindered organolithium reagents. Presumably, the reaction proceeds via SET from the organometallic compound and iminoxyl radical with the formation of an oxime anion and an intermediate C-centered radical. In the case of MeLi and PhLi, which correspond to the most reactive methyl and phenyl radicals, products **44** of hydrogen atom abstraction from Et_2_O followed by C–O coupling of the resultant C-centered radical with the iminoxyl radical was observed.

**Scheme 15:**
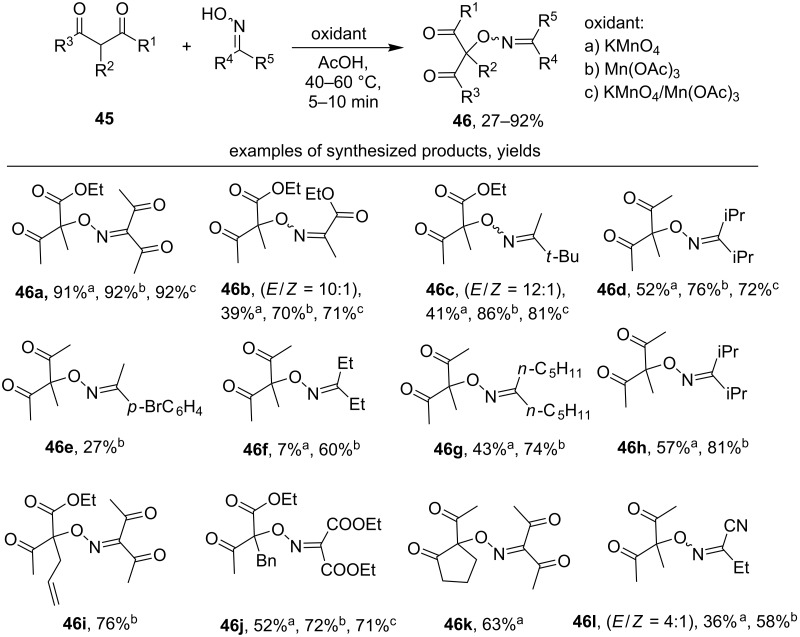
Cross-dehydrogenative C–O coupling of 1,3-dicarbonyl compounds with oximes under the action of manganese-based oxidants.

Recently, examples of selective intermolecular C–O coupling between oxime radicals generated in situ from oximes and different types of CH-reagents have been reported. 1,3-Diketones and 1,3-ketoesters **45** undergo cross-dehydrogenative C–O coupling with oximes under the action of oxidizing agents [46], such as KMnO_4_, Mn(OAc)_3_ or the KMnO_4_/Mn(OAc)_3_ system (Scheme 15). A radical mechanism is suggested for the formation of the C–O coupling products **46** in which the oxidizing agent serves to generate oxime radicals from oximes and perform one-electron oxidation of 1,3-dicarbonyl compounds **45**. The formation of oxime radicals under the reaction conditions was confirmed by EPR spectroscopy [46].

1,3-Diketones and 1,3-ketoesters with easily oxidizable groups, such as allyl and benzyl, were tolerated (**46i** and **46j**). The yield of the C–O cross-coupling products **46** increases with the rise in the stability of the corresponding oxime radicals. For example, in the row **46f–h** the yield becomes higher with the increase of the steric effect of the alkyl substituents attached to the oxime group. The lowest yield was obtained with an aromatic oxime (product **46e**, yield 27%).

**Scheme 16:**
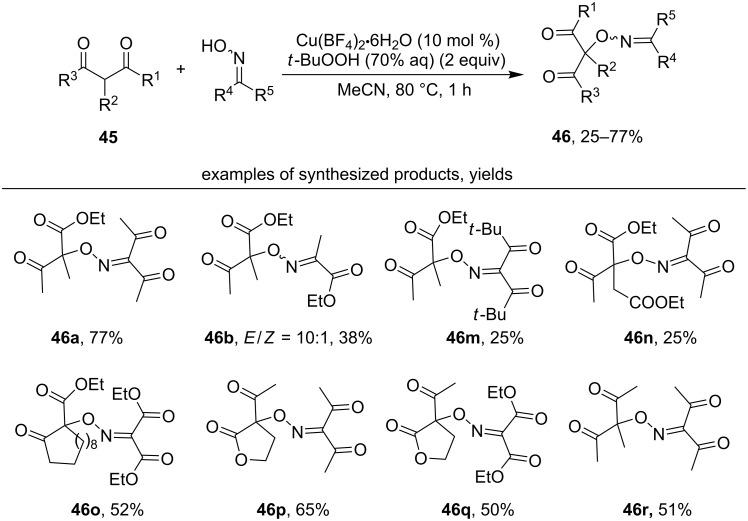
Cross-dehydrogenative C–O coupling of 1,3-dicarbonyl compounds with oximes under the action of Cu(BF_4_)_2_ (cat.)/*t*-BuOOH system.

Later, the Cu(BF_4_)_2_ (cat.)/*t*-BuOOH oxidative system [87] was proposed as an alternative to stoichiometric metal-containing oxidants, such as KMnO_4_, Mn(OAc)_3_, and KMnO_4_/Mn(OAc)_3_ (Scheme 16).

1,3-Ketoesters (products **46a**,**b**, **46m**,**n**), 1,3-diketones (product **46r**), as well as lactones (products **46o–q**) were used in the oxidative C–O coupling reaction. The coupling of oximes with 1,3-diketones proceeded in lower yields than with 1,3-ketoesters (products **46a** and **46r**). Despite the presence of *t*-BuOOH in the system, a Kharash peroxidation of 1,3-dicarbonyl compounds [88–93] did not occur, and a selective formation of the C–O product with oximes was observed.

**Scheme 17:**
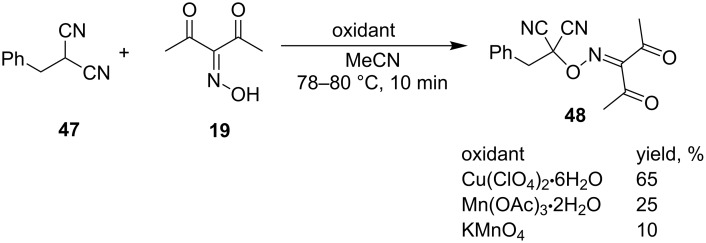
Oxidative C–O coupling of benzylmalononitrile (**47**) with 3-(hydroxyimino)pentane-2,4-dione (**19**).

Benzylmalononitrile (**47**) was introduced into the oxidative C–O coupling with diacetyl oxime (**19**) analogously to 1,3-dicarbonyl compounds [46], but Cu(ClO_4_)_2_ afforded a better yield of the C–O coupling product **48** then the manganese-based oxidants in this case (Scheme 17) [94].

**Scheme 18:**
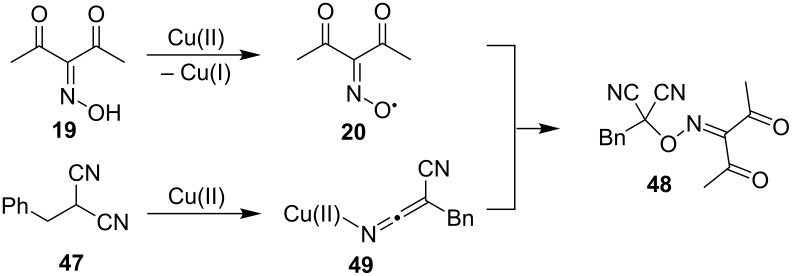
The proposed mechanism of the oxidative coupling of benzylmalononitrile (**47**) with diacetyl oxime (**19**).

A radical mechanism was suggested. The copper(II) ion reacts with oxime **19** to generate iminoxyl radical **20** and also forms complex **49** with dinitrile **47**. Interaction of radical **20** and complex **49** results in the coupling product **48** (Scheme 18). The formation of radical **20** from the oxime **19** under the action of Cu(ClO_4_)_2_ in acetonitrile was proved by EPR spectroscopy [46].

**Scheme 19:**
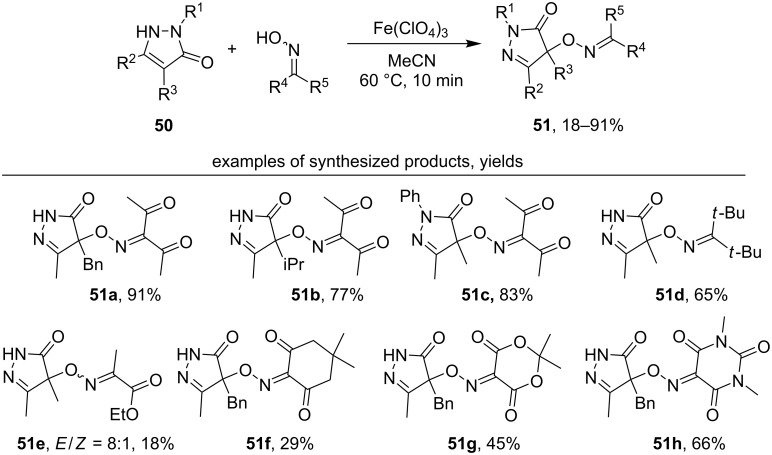
Oxidative C–O coupling of pyrazolones with oximes under the action of Fe(ClO_4_)_3_.

Free-radical oxidative C–O coupling of pyrazolones **50** with different classes of *N*-hydroxy compounds, including oximes, was demonstrated [44]. In contrast to the cross-dehydrogenative coupling of oximes with 1,3-dicarbonyl compounds, both one-electron oxidants (Fe(ClO_4_)_3_, (NH_4_)_2_Ce(NO_3_)_6_) and two-electron oxidants (PhI(OAc)_2_, Pb(OAc)_4_), that vary greatly in properties, are applicable for this process. After optimization of the reaction conditions Fe(ClO_4_)_3_ was chosen as the optimal oxidant for the synthesis of C–O cross-dehydrogenative coupling products **51** (Scheme 19).

**Scheme 20:**
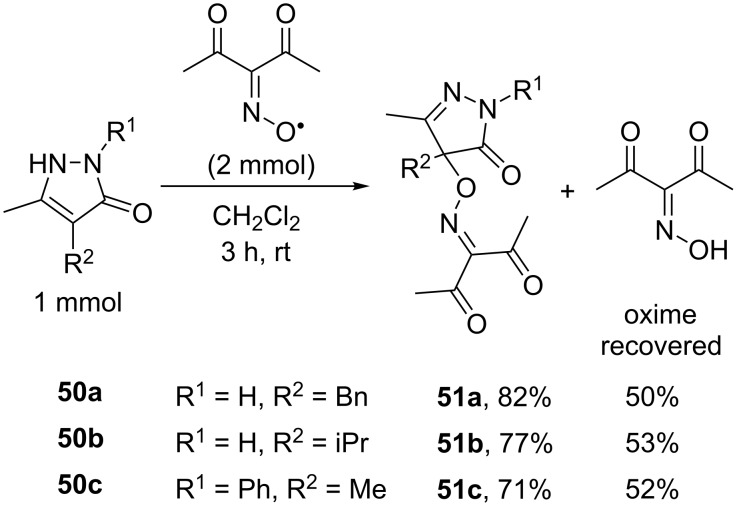
The reaction of diacetyliminoxyl radical with pyrazolones.

The extremely persistent diacetyliminoxyl radical (**20**) [44] was directly introduced into the reaction with pyrazolones **50** with the formation of the corresponding C–O coupling products **51** (Scheme 20). The yields were close to that obtained with in situ generated of iminoxyl radicals (Scheme 19).

**Scheme 21:**
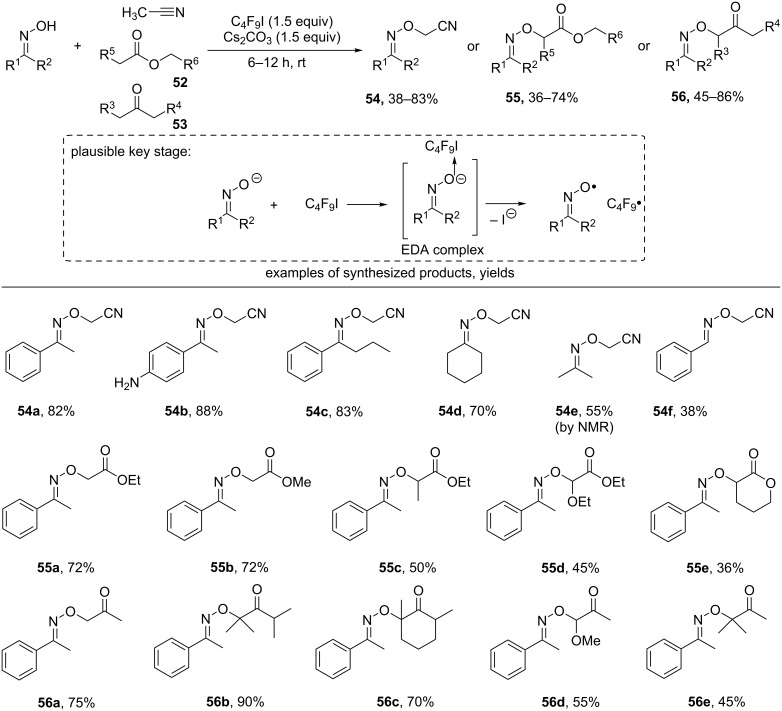
Oxidative C–O coupling of oximes with acetonitrile, ketones, and esters.

Recently, the oxidative C–O coupling of oximes with acetonitrile, esters **52**, and ketones **53** was realized [95] (Scheme 21). The authors suggested a radical mechanism in which the iminoxyl radical is generated from the oxime anion under the action of perfluorobutyl iodide through the formation of an EDA complex (electron donor–acceptor complex, which is also called charge-transfer complex). The perfluorobutyl radical formed at this step served for the hydrogen atom abstraction from the CH-reagent (MeCN, **52** or **53**).

Ketone oximes of both aromatic (products **54a–c**) and aliphatic structures (**54d**,**e**) were successfully used in coupling with acetonitrile. The C–O coupling product **54f** of acetonitrile with benzaldoxime was obtained in a lower yield. The aromatic oximes reacted with esters and ketones to give oxidative coupling products in moderate to good yields (products **55a–e** and **56a–e**, respectively). In the case of asymmetric ketones, the C–H bond at the more substituted carbon was cleaved (products **56d**,**e**).

Recently, the copper-catalyzed addition of oximes to the C=C double bond of maleimides was reported [96]. The iminoxyl radicals were detected by EPR spectroscopy, but the non-radical mechanism (copper-catalyzed Michael addition) can not be excluded completely.

### Application of the oxime radicals in organic synthesis: intramolecular reactions

**Scheme 22:**
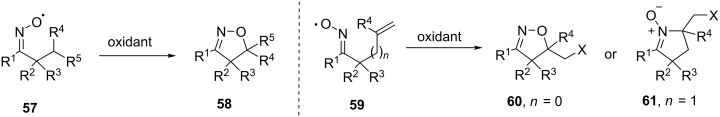
Intramolecular cyclizations of oxime radicals to form substituted isoxazolines or cyclic nitrones.

There are two main types of intramolecular reactions involving oxime radicals (Scheme 22). In the first type, an initial hydrogen atom abstraction is followed by a cyclization (transformation of **57** to **58**). In the second type, an addition of oxime radicals to a C=C double bond takes place (transformation of **59** to **60** or **61**). As a result of the reaction, a 5-membered cycle is formed via the formation of C–O (products **58** and **60**) or C–N bond (product **61**) in accordance with the ability of oxime radicals to act as O- or N-radicals.

The formation of the heterocycles, mainly isoxazolines/isoxazoles, from unsaturated oximes can be achieved through different ways including addition of electrophiles to the C=C double bond of the unsaturated oxime followed by intramolecular nucleophilic attack of the oxime group [97–100], metal-catalyzed cyclization [98,101–108], cyclization under the action of photocatalysts [109–110], cyclization of nitroso intermediate [111], etc. [112–113]. At least some of these reactions do not involve oxime radicals as intermediates. It should be noted that free-radical cyclizations mediated by iminoxyl radicals frequently afford products that are hardly achievable or not achievable by non-radical methods. In this review only works in which the participation of iminoxyl radicals was confirmed or assumed are discussed.

#### Oxidative cyclization with the cleavage of the C–H bond

**Scheme 23:**
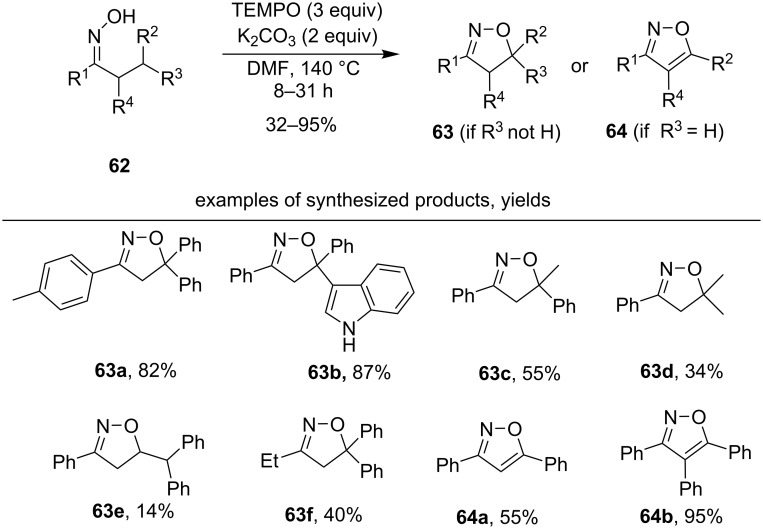
TEMPO-mediated oxidative cyclization of oximes with C–H bond cleavage.

In one of the first works in this area oximes with activated C–H bond in the β-position were transformed into isoxazolines or isoxazoles by oxidative cyclization [114] under the action of TEMPO and K_2_CO_3_ (Scheme 23).

The presence of aryl substituents at the β-position of the oxime contributed to high yields of the desired products (**63a–c**, 55–87%), in the presence of only methyl substituents moderate yields were observed (**63d**, 34%). The reaction proceeds exclusively with the closure of the five-membered cycle and participation of the C–H bond located exclusively in the β-position with respect to the oxime group. This regularity is maintained even when the activated benzylic C–H bond is present in the γ-position with respect to the oxime group (example **63e**, yield 14%). Almost in all examples, an aryl substituent (R^1^ = Ph or substituted phenyl) was located at the oxime group; the product **63f** with R^1^ = Et was obtained in a moderate yield of 40%. In the presence of only one aryl group in the β-position (R^3^ = H, R^2^ = Ar) and further processing of the reaction mixture with atmospheric oxygen, an aromatization occurs with the formation of isoxazoles (**64a,b** 55–95%).

**Scheme 24:**
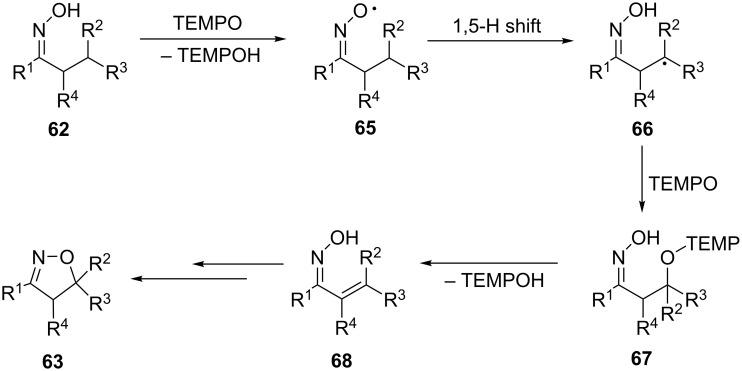
Proposed reaction mechanism of oxidative cyclization of oximes with C–H bond cleavage.

Presumably, the reaction of TEMPO with oxime **62** affords the iminoxyl radical **65** (Scheme 24). 1,5-HAT in the radical **65** gives a C-centered radical **66**, which is captured by TEMPO to form intermediate **67**. Elimination of TEMPOH leads to a β-unsaturated oxime **68**, which could undergo cyclization by ionic or radical mechanisms to give isoxazoline **63** [114–115].

**Scheme 25:**
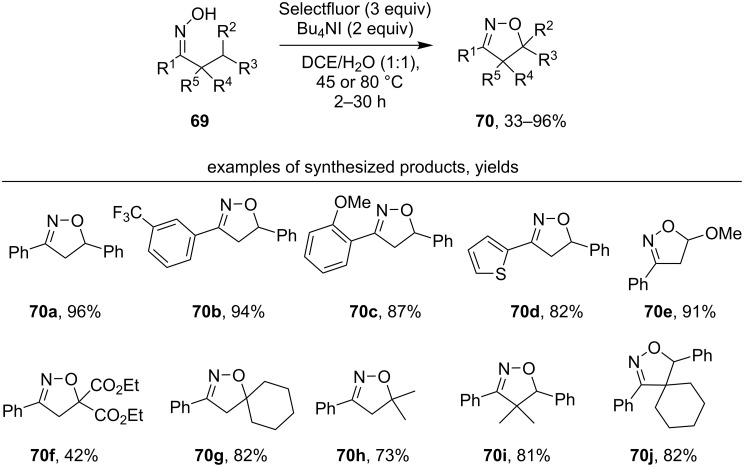
Selectfluor/Bu_4_NI-mediated C–H oxidative cyclization of oximes.

A similar cyclization with the formation of isoxazolines **70** was realized [116] by the oxidation of oximes **69** by the Selectfluor/Bu_4_NI system (Scheme 25). A radical mechanism was proposed in which the hypoiodite formed from the oxime undergoes a homolytic cleavage of the O–I bond with the formation of the iminoxyl radical.

As a rule, R^1^ is an aromatic ring, and the yield of the target product weakly depends on the electronic effects of substituents in this ring (products **70a–d**). Good yields were obtained even with substrates having inert non-benzyl C(sp^3^)–H bonds (products **70f–h**). It is important to note that products with two substituents in the α-position to the oxime group (**70i**,**j**) were obtained with good yields. The formation of **70i** and **70j** is impossible through an intermediate similar to intermediate **68** in Scheme 24.

**Scheme 26:**
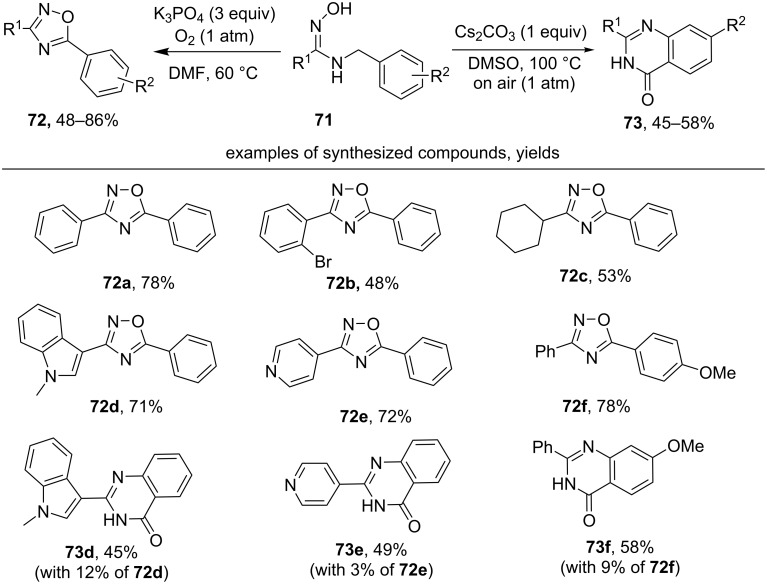
Oxidative cyclization of *N*-benzyl amidoximes to 1,2,4-oxadiazoles.

Oxidative cyclization of *N*-benzyl amidoximes **71** was realized [117] under the action of molecular oxygen with the formation of either 1,2,4-oxadiazoles **72** or quinazolinones **73** (Scheme 26), depending on the reaction conditions. The 1,2,4-oxadiazole ring was selectively obtained in DMF at 60 °С under oxygen atmosphere (1 atm) in the presence of an excess of K_3_PO_4_, whereas in DMSO at 100 °С under air and in the presence of Cs_2_CO_3_ quinazolinones **73** were selectively synthesized.

**Scheme 27:**
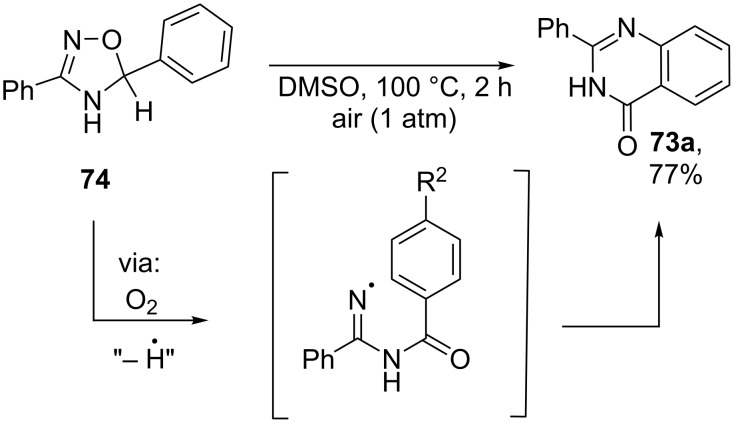
The formation of quinazolinone **73a** from 5-phenyl-4,5-dihydro-1,2,4-oxadiazole **74** under air.

The authors proposed that 1,2,4-oxadiazoles were formed by a mechanism [117], analogous to the mechanism of the TEMPO-mediated oxidative oxime cyclization (Scheme 23 [114]). Apparently, both 1,2,4-oxadiazoles **72** and quinazolinones **73** are produced via the common intermediate, 4,5-dihydro-1,2,4-oxadiazole. An example of such intermediate **74** is shown in Scheme 27. Oxidative aromatization of **74** leads to 1,2,4-oxadiazole **72a** (Scheme 26). The second pathway, hydrogen abstraction followed by β-scission presumably leads to iminyl radical, which forms the observed quinazolinone **73a** (Scheme 27) [118].

**Scheme 28:**
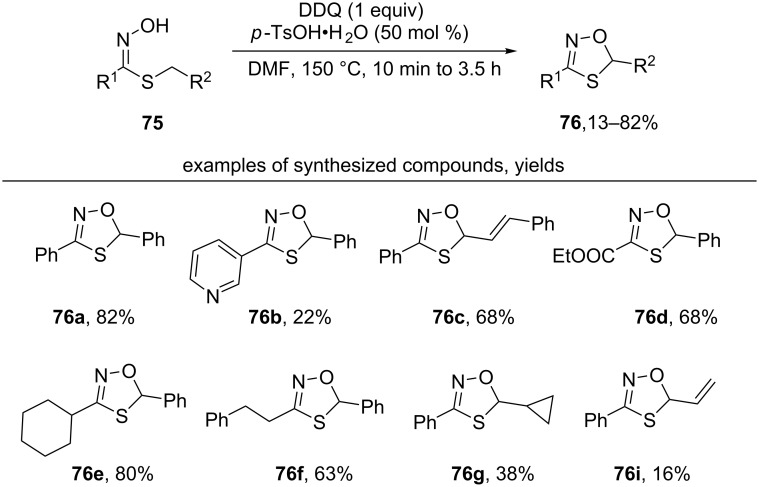
DDQ-mediated oxidative cyclization of thiohydroximic acids.

The method for oxidative cyclization of thiohydroximic acids **75** under the action of DDQ and *p*-TsOH with the formation of the corresponding 1,4,2-oxathiazoles **76** was developed (Scheme 28) [119]. The authors noted that reaction in the absence of *p*-TsOH proceeded with lower yield of **76**.

**Scheme 29:**
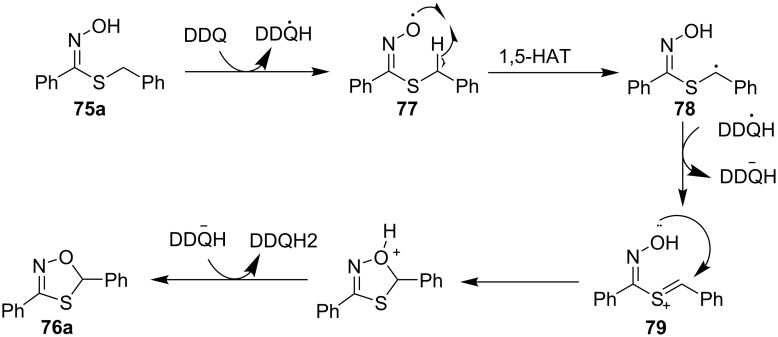
Plausible mechanism of the oxidative cyclization of thiohydroximic acids.

A radical mechanism was proposed in which the oxime moiety is oxidized by DDQ to the iminoxyl radical **77**, which undergoes 1,5-HAT to give a C-centered radical **78** stabilized by a sulfur atom. **78** is oxidized by DDQ to a carbocation **79**, followed by the closure of the oxathiazole ring (Scheme 29). Later, DDQ-mediated oxidative cyclization of amidoximes with the formation of 1,2,4-oxadiazoles (analogous transformation with K_3_PO_4_/O_2_ system was shown above in Scheme 26) was realized without the addition of TsOH [120].

**Scheme 30:**
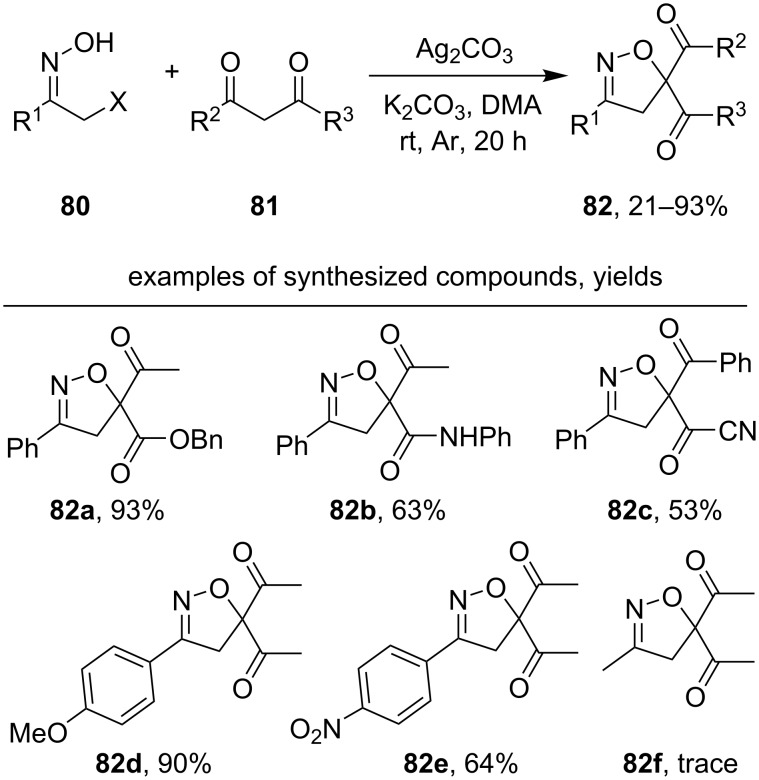
Silver-mediated oxidative cyclization of α-halogenated ketoximes and 1,3-dicarbonyl compounds.

Isoxazolines **82** were synthesized by a one-pot sequence, which included the substitution of a halogen atom in α-halogenated oxime **80** by dicarbonyl compound **81** and oxidative cyclization (Scheme 30) [121].

**Scheme 31:**
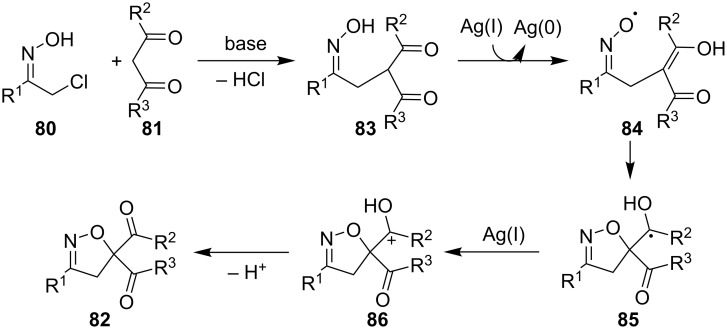
Possible pathway of one-pot oxidative cyclization of α-halogenated ketoximes and 1,3-dicarbonyl compounds.

The introduction of electron-donating substituents into the benzene ring of the oxime increases the yield of the reaction product (example **82d**, 90%), and the introduction of the electron-withdrawing substituents decreases it (example **82e**, 64%). Non-aromatic oximes of chloro- or bromoacetone are not suitable for this method; product **82f** was observed in trace amounts. According to the proposed reaction pathway, the nucleophilic substitution of the halogen atom with the formation of intermediate **83** proceeds in the presence of a base (Scheme 31). Oxidation of **83** with silver(I) affords the iminoxyl radical **84**, which undergoes cyclization to form **85**. Subsequent oxidation leads to intermediate **86**, which is deprotonated to form the final product **82**.

**Scheme 32:**
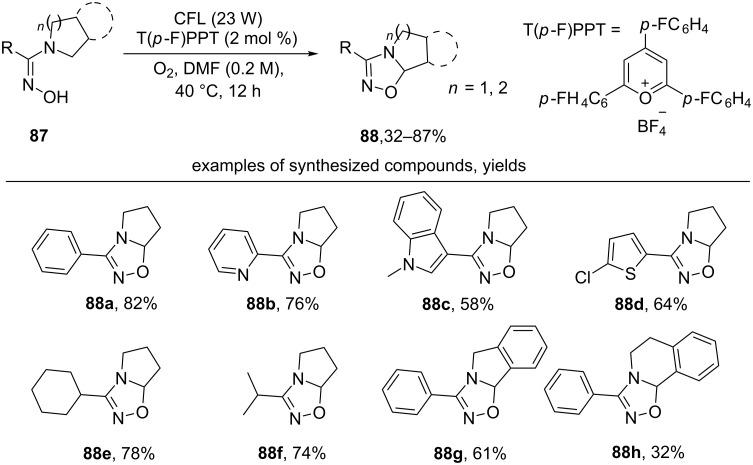
T(*p*-F)PPT-catalyzed oxidative cyclization of oximes with the formation of 1,2,4-oxadiazolines.

A convenient method for the synthesis of 1,2,4-oxadiazolines **88** by oxidative cyclization of amidoximes **87** under the action of molecular oxygen and visible light in the presence of catalytic amounts of 2,4,6-tris(4-fluorophenyl)pyrilium tetrafluoroborate (T(*p*-F)PPT) was proposed (Scheme 32) [122].

Pyrrolidinyl oxime derivatives having both aromatic (products **88a–d**) and aliphatic (products **88e**,**f**) substituents are applicable. Oximes with an isoindoline or tetrahydroisoquinoline fragment also undergo this transformation to give substituted oxadiazolines (products **88g**,**h**). The authors note that T(*p*-F)PPT plays the role of a photocatalyst that promotes the formation of an oxime radical that undergoes 1,5-HAT to form the target product.

#### Oxidative cyclization with the cleavage of π-bond C=C

**Scheme 33:**
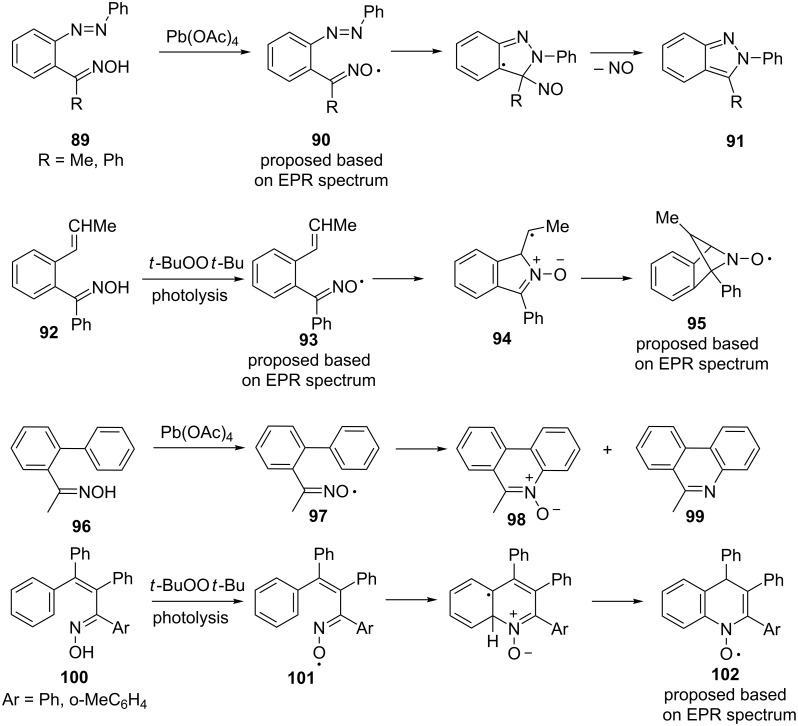
Intramolecular cyclization of iminoxyl radicals involving multiple C=C and N=N bonds.

Early examples of oxidative cyclization of iminoxyl radicals with an attack on π-bonds were reported in the 1980s [123]. However, the structure of the products was not exactly proved, the scope of application and preparative potential of these reactions was not studied (Scheme 33).

When the oxime **89** with an azo fragment was treated by lead(IV) acetate at −60 °C, an EPR signal with the HFS constant a^N^ = 31 G was observed which indicated the formation of the iminoxyl radical **90**. Presumably, the radical **90** underwent cyclization involving the azo group to form indazole **91**.

During the photolysis of a mixture of di-*tert*-butyl peroxide with oxime **92** containing an alkenyl fragment at temperatures from −30 to −10 °C, two signals were observed in the EPR spectrum with a constant HFC a^N^ = 30 G and 32 G, corresponding to iminoxyl radicals **93**. At higher temperature (+10 °C), only one signal was observed with a^N^ = 19.75 G. This HFS value is characteristic of bicyclic nitroxyl radicals [123]. The authors suggested that the formed iminoxyl radical underwent cyclization with the formation of alkyl radical **94**. The latter attacked the nitrone moiety to form the bicyclic nitroxyl radical **95**. When oxime **96** was oxidized with lead(IV) acetate, products **98** and **99** were observed. This result can be explained by the intramolecular attack of iminoxyl radical **97** on the phenyl π-system. According to EPR data, the authors suggested that iminoxyl radicals **101** generated from oximes **100** by photolysis with the addition of the di-*tert*-butyl peroxide gave nitroxides **102** [123].

**Scheme 34:**
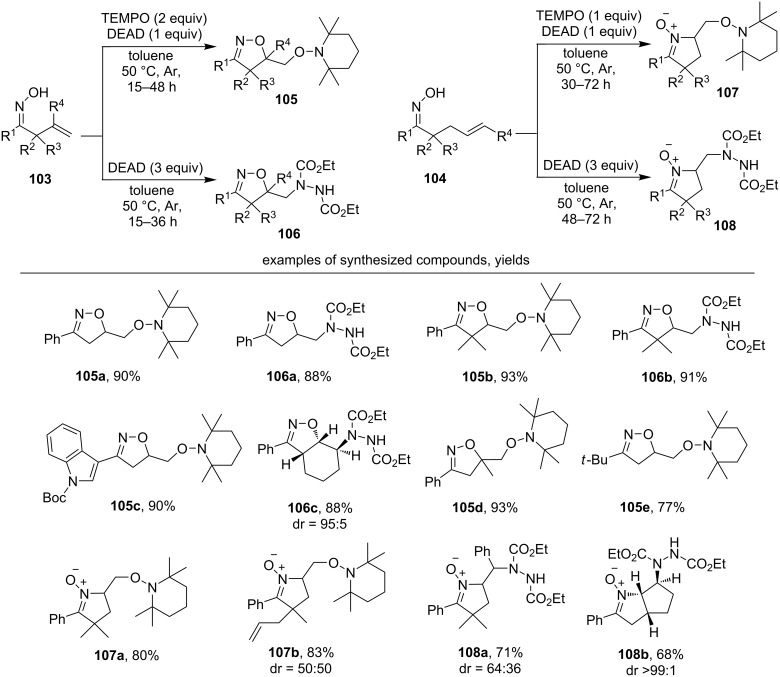
Oxidative cyclization of β,γ- and γ,δ-unsaturated oximes employing the DEAD or TEMPO/DEAD system with the formation of C–O and C–N bonds.

The widespread use of iminoxyl radicals in organic synthesis involving a radical addition to a C=C bond started to develop extensively only after 2010. The oxidation of β,γ- and γ,δ-unsaturated oximes (**103** and **104**, respectively) using the TEMPO/DEAD system or only DEAD (diethyl azodicarboxylate) afforded 5-*exo-trig* radical cyclization [124] with the formation of the corresponding isoxazolines **105** and **106** or cyclic nitrones **107** and **108** (Scheme 34).

A variety of β,γ-unsaturated oximes **103** with an aromatic substituent at the C=NOH group reacted with the formation of isoxazolines (products **105a–d**, **106a–c**, yields 88–93%). Product **105e** containing a non-aromatic *tert*-butyl R^1^ group was synthesized in good yield (77%). When γ,δ-unsaturated oximes were applied the formation of cyclic nitrones was observed (products **107a**,**b, 108a**,**b**). In this case, the intermediate oxime radicals reacted as N-centered radicals, which was consistent with the calculations [124]. If the double bond of the starting oxime was endocyclic, high stereoselectivity was observed with the formation of *trans*-products (examples **106c** and **108b**).

**Scheme 35:**
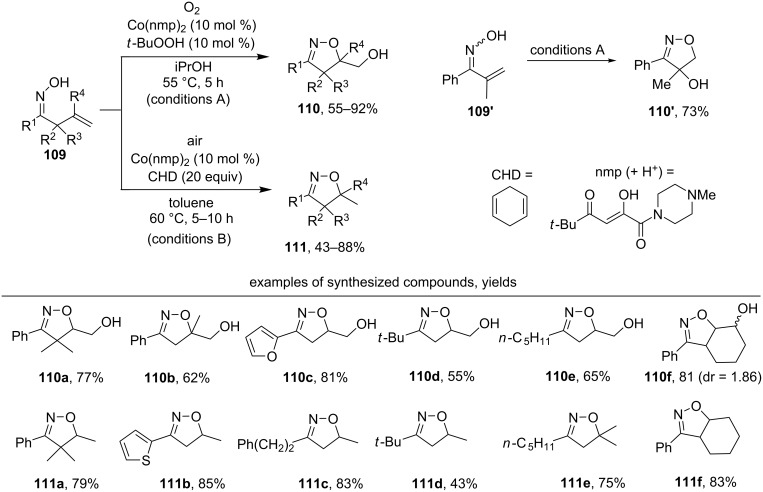
Cobalt-catalyzed aerobic oxidative cyclization of β,γ-unsaturated oximes.

The oxidative cyclization of β,γ-unsaturated oximes **109** under the action of molecular oxygen and catalytic amounts of bis(5,5-dimethyl-1-(4-methylpiperazin-1-yl)hexane-1,2,4-trione)cobalt(II) (Co(nmp)_2_) resulted in isoxazolines **110** with a hydroxylmethyl group or methylisoxazolines **111** (Scheme 35) [125].

The reaction in iPrOH under an oxygen atmosphere with the addition of 10 mol % of *t*-BuOOH (conditions A) produced hydroxymethylisoxazolines **110**, and the reaction in toluene under air with the addition of 20 equivalents of cyclohexa-1,4-diene (CHD) as hydrogen atom donor (conditions B) led to methylisoxazolines **111**. Both aromatic (examples **110a–c**,**f**, **111a**,**b**,**f**) and aliphatic (examples **110d**,**e** and **111c–e**) β,γ-unsaturated oximes undergo this cyclization. The conditions A were also applied for the oxidation of α,β-unsaturated oxime **109’**. As a result, 5-*endo-trig* cyclization affording hydroxyisoxazoline **110’** in good yield was observed.

**Scheme 36:**
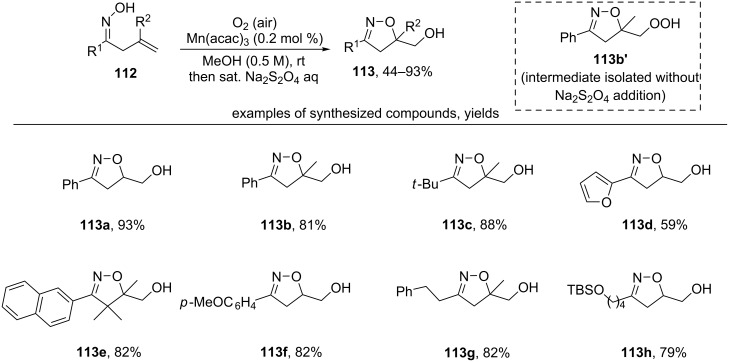
Manganese-catalyzed aerobic oxidative cyclization of β,γ-unsaturated oximes.

Another approach to the synthesis of hydroxy-substituted isoxazolines **113** is the manganese(III) acetylacetonate catalyzed reaction of β,γ-unsaturated oximes **112** with oxygen of air (Scheme 36) [126].

The peroxide initially formed in the reaction was reduced by treatment with a saturated Na_2_S_2_O_4_ solution. The formation of peroxide was confirmed by a control experiment in which the hydroperoxide **113b’** was isolated when the treatment of the reaction mixture with sodium dithionite was omitted. High yields were reached for both monosubstituted (products **113a**,**d**,**f**,**h**) and disubstituted C=C bonds (products **113b**,**c**,**e**,**g**). Aliphatic oximes also undergo this transformation in high yields (products **113c**,**g**,**h**).

**Scheme 37:**
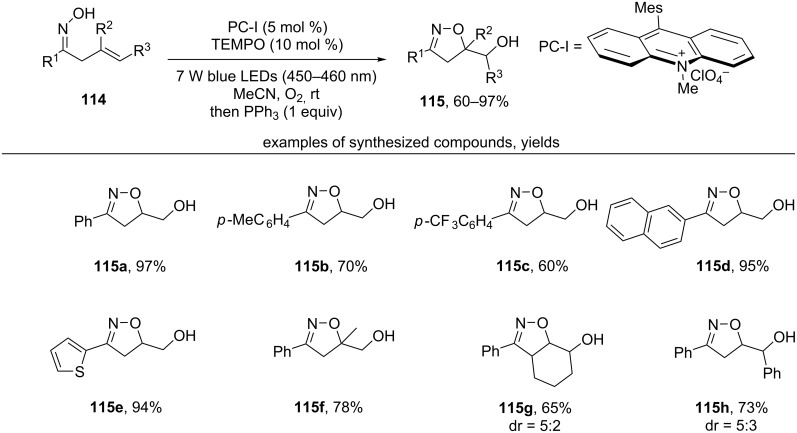
Visible light photocatalytic oxidative cyclization of β,γ-unsaturated oximes.

The photocatalytic oxidative cyclization of β,γ-unsaturated oximes **114** was carried out under the action of a PC-I/TEMPO catalytic system and the irradiation of blue LEDs (Scheme 37) [127].

A variety of electronically rich and electronically poor aryl or heteroaryl groups at the oxime group (R^1^) are well-tolerated (products **115a–e**). Oximes with disubstituted double C=C bond also successfully undergo this cyclization (products **115f–h**).

**Scheme 38:**
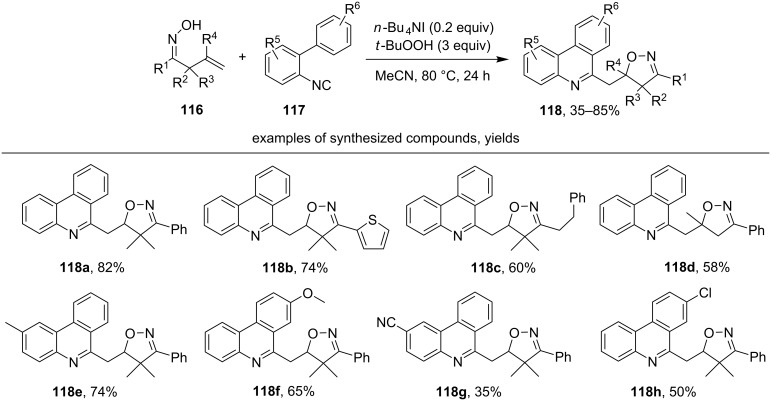
TBAI/TBHP-mediated radical cascade cyclization of the β,γ-unsaturated oximes.

C-centered radicals generated in a radical 5-*exo-trig* cyclization of β,γ-unsaturated oximes **116** upon oxidation by the TBAI/TBHP system were trapped by an isonitrile group of 2-arylphenyl isonitriles **117** to synthesize substituted phenanthridines **118** (Scheme 38) [128].

Both aliphatic (example **118c**) and aromatic (examples **118a**,**b**,**d–h**) β,γ-unsaturated oximes enter this cascade cyclization. Relatively low yields were obtained with electron-withdrawing substituents in isonitrile **117** (examples **118g**,**h**).

**Scheme 39:**
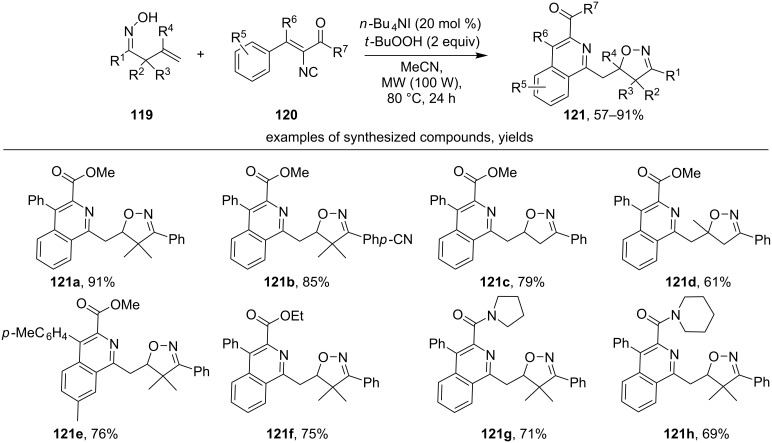
TBAI/TBHP-mediated radical cascade cyclization of vinyl isocyanides with β,γ-unsaturated oximes.

Another example of a cascade oxidative cyclization involving an isonitrile group is the reaction of β,γ-unsaturated oximes **119** with vinyl isocyanides **120** to form substituted isoquinolines **121** (Scheme 39) [129].

Both α,α-disubstituted aromatic oximes (products **121a**,**b**,**e–h**) and unsubstituted (R^2^ = R^3^ = H, products **121c**,**d**) undergo the reaction successfully. The authors noted the effect of substituents in the phenyl fragment attached to the oxime group (R^1^): high yields were obtained with *para*- and *meta*-substituted substrates; however, when *ortho*-substituents were present, the product **121** was not observed. Two aryl groups at the vinyl fragment of the isocyanide are important for a successful synthesis of products **121**. In the case of R^6^ = H or alkyl the formation of the oxidative cyclization product **121** was not observed [129].

**Scheme 40:**
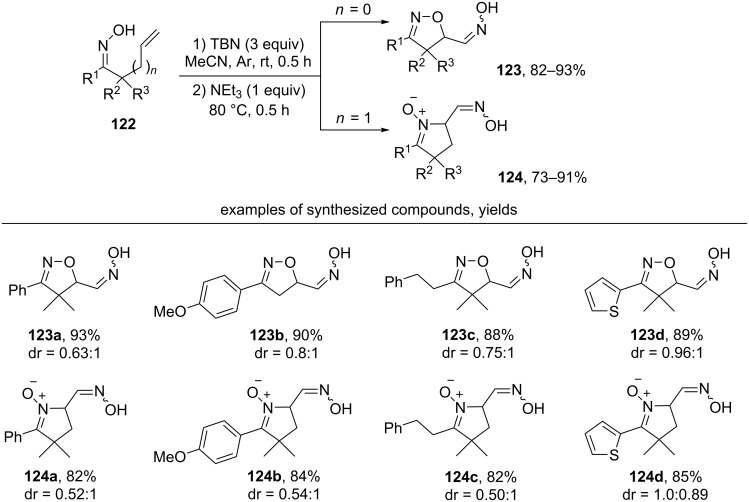
*tert*-Butylnitrite-mediated oxidative cyclization of unsaturated oximes with the introduction of an additional oxime group.

The oxidative cyclization of unsaturated oximes **122** under the action of *t*-BuONO (TBN), followed by treatment with NEt_3_ leads to isoxazolines **123** or cyclic nitrones **124** with an additional oxime group (Scheme 40) [130].

**Scheme 41:**
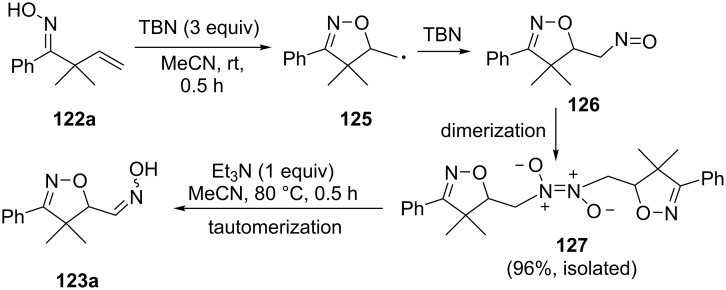
Transformation of unsaturated oxime to oxyiminomethylisoxazoline via the confirmed dimeric nitroso intermediate.

The authors showed that the initial product of the oxidative cyclization of oxime **122a** under the action of TBN was the dimer **127** of the nitroso compound **126**, which was formed, presumably, as a result of nitrosation of the C-centered radical **125** by TBN [130]. Intermediate **127** was isolated in 96% yield and its structure was confirmed by a single-crystal X-ray diffraction. Under the action of Et_3_N, the dimeric nitroso compound **127** was converted into the more stable oxime tautomeric form **123a** (Scheme 41).

**Scheme 42:**
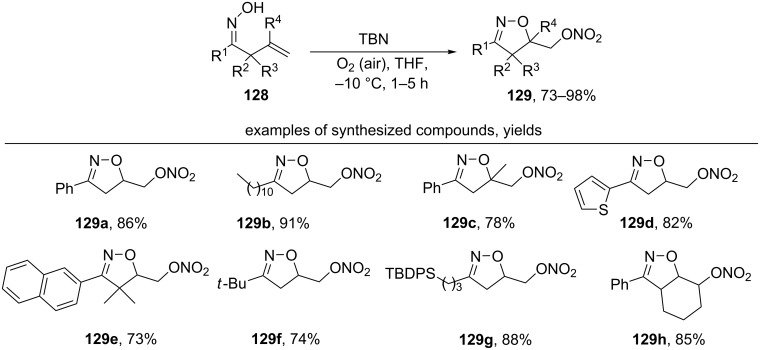
*tert*-Butylnitrite-mediated oxidative cyclization of unsaturated oximes with the introduction of a nitrooxy group.

Another reaction pathway of a TBN-mediated oxidative cyclization of β,γ-unsaturated oximes **128** was achieved by switching from the argon atmosphere to air or oxygen atmosphere and by lowering the reaction temperature to –10 °C [131]. Oximes **128** undergo cyclization to form nitrooxymethyl-substituted isoxazolines **129** (Scheme 42). THF was found to be the optimal solvent.

Aliphatic (examples **129b**,**f**,**g**) and aromatic (examples **129a**,**c**,**d**,**e**,**h**) unsaturated oximes undergo this cyclization. Oximes containing disubstituted double bonds also give the corresponding isoxazolines **129c**,**h**. The authors proposed a mechanism involving a 5-*exo-trig* cyclization of the oxime radical followed by the addition of molecular oxygen to the formed C-centered radical with a formation of a peroxyl radical. The interaction of the latter with NO leads to the final oxynitro compound [131].

**Scheme 43:**
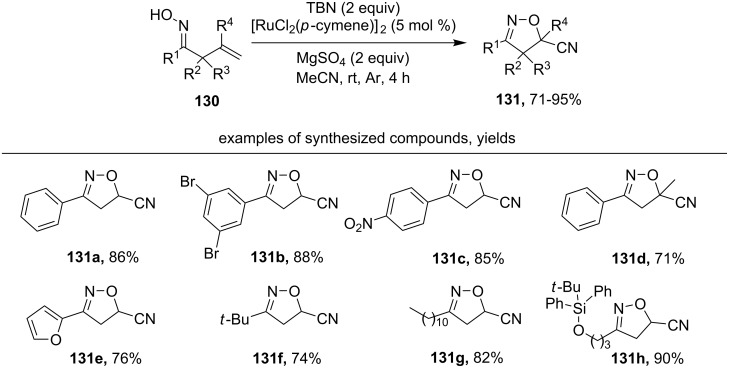
Synthesis of cyano-substituted oxazolines from unsaturated oximes using the TBN/[RuCl_2_(*p*-cymene)]_2_ (cat.) system.

Cyano-substituted oxazolines **131** were synthesized from unsaturated oximes **130** using a combination of *t*-BuONO and a ruthenium catalyst (Scheme 43) [132]. The authors proposed that the interaction of unsaturated oxime with TBN produced a hydroxyiminomethylisoxazoline (Scheme 40) [130] that was transformed into the cyano-substituted oxazoline in the presence of a ruthenium catalyst. This possible reaction pathway was confirmed by a control experiment in which the hydroxyiminomethylisoxazoline was transformed to a nitrile in the presence of [RuCl_2_(*p*-cymene)]_2_.

Aromatic oximes with various substituents, as well as heteroaromatic oximes, give cyano-substituted oxazolines in good yields (products **131a–e**). Aliphatic oximes also enter this transformation, including an oxime containing a TBDPS protecting group (products **131f–h**).

**Scheme 44:**
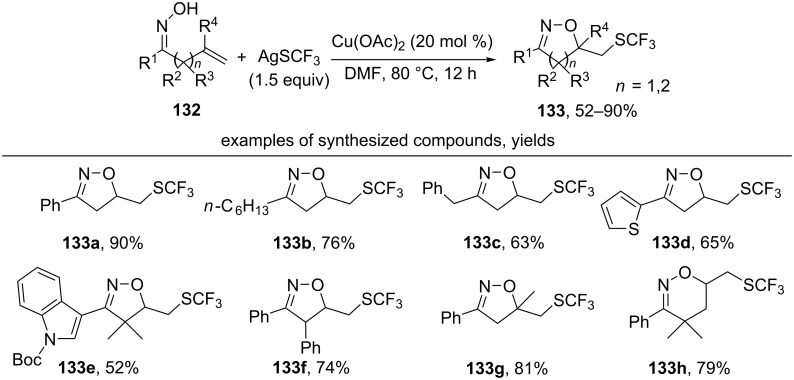
Synthesis of trifluoromethylthiolated isoxazolines from unsaturated oximes.

The combination of AgSCF_3_ and catalytic amounts of Cu(OAc)_2_ was used for the synthesis of trifluoromethylthiolated isoxazolines **133** from unsaturated oximes **132** (Scheme 44) [133].

Substrates with both aryl (products **133a**,**d–h**) and alkyl substituents (product **133b**) at the oxime fragment (R^1^ in **132**) were successfully used for the oxidative cyclization. The proposed reaction mechanism involves the formation of an oxime radical and its 5-*exo-trig* cyclization to form a C-centered radical, which undergoes a trifluoromethylthiolation by •SCF_3_-radical-generated from AgSCF_3_ [133]. The iminoxyl radical 5-*exo-trig* cyclization step was confirmed in experiments with the capture of a C-centered radical by TEMPO. It should be noted that in the case of γ,δ-unsaturated oxime an unusual six-membered oxazine **133h** was reported as the major product [133].

**Scheme 45:**
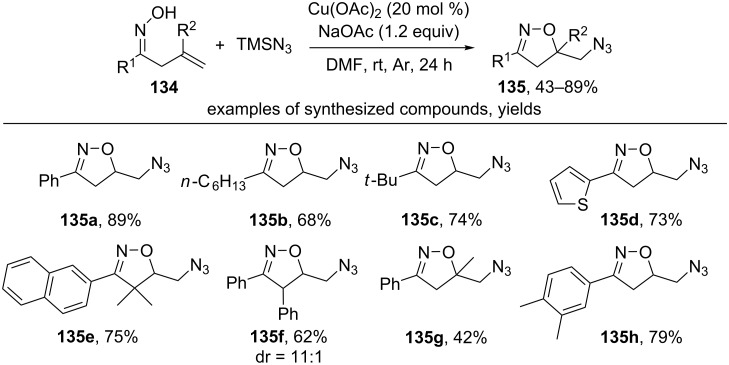
Copper-сatalyzed oxidative cyclization of β,γ-unsaturated oximes with the introduction of an azido group.

A similar cyclization of oximes **134** with the introduction of an azido group was carried out using TMSN_3_ as an azide source (Scheme 45) [134].

The reaction is applicable for β,γ-unsaturated oximes having both aryl (products **135a,d–h**) and alkyl substituents (products **135b,c**) at the oxime fragment (R^1^). An oxime with a disubstituted double bond (R^2^ = Me) also reacts with the formation of isoxazoline **135g** having a quaternary carbon atom.

**Scheme 46:**
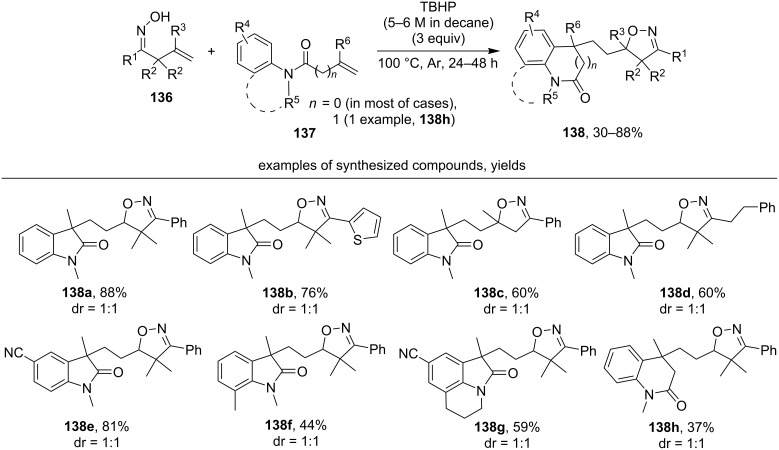
TBHP-mediated oxidative cascade cyclization of β,γ-unsaturated oximes and unsaturated *N*-arylamides.

Under the action of *t*-BuOOH (TBHP), β,γ-unsaturated oximes **136** undergo a cascade cyclization with *N*-aryl-*N*-methylmethacrylamides **137** affording substituted oxoindoles **138** (Scheme 46) [135].

In the majority of examples, aromatic β,γ-unsaturated oximes (examples **138a–c, e–h**) were used. Oximes having a disubstituted double bond also successfully entered this reaction (example **138c**). The relatively low yield of product **138f** was explained by the steric effect of the *ortho*-methyl substituent in amide **137**. In most cases, *N*-aryl-*N*-methylmethacrylamides were used for this cyclization to obtain oxindoles (products **138a–g**) except for one example where a homologous amide was used to obtain six-membered lactam **138h**.

**Scheme 47:**
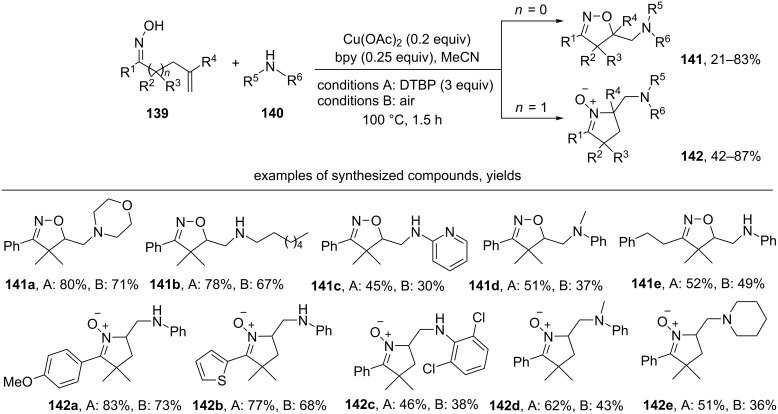
Copper-сatalyzed oxidative cyclization of unsaturated oximes with the introduction of an amino group.

The catalytic system Cu(OAc)_2_/bipyridine (bpy) was applied to perform the oxidative cyclization of unsaturated oximes **139** accompanied by the introduction of an amino group with the formation of isoxazolines **141** and cyclic nitrones **142** (Scheme 47) [136].

The best results were obtained using DTBP or aerial oxygen as an oxidant. Aliphatic, aromatic, and heteroaromatic amines **140**, both primary and secondary, are applicable for this reaction.

**Scheme 48:**
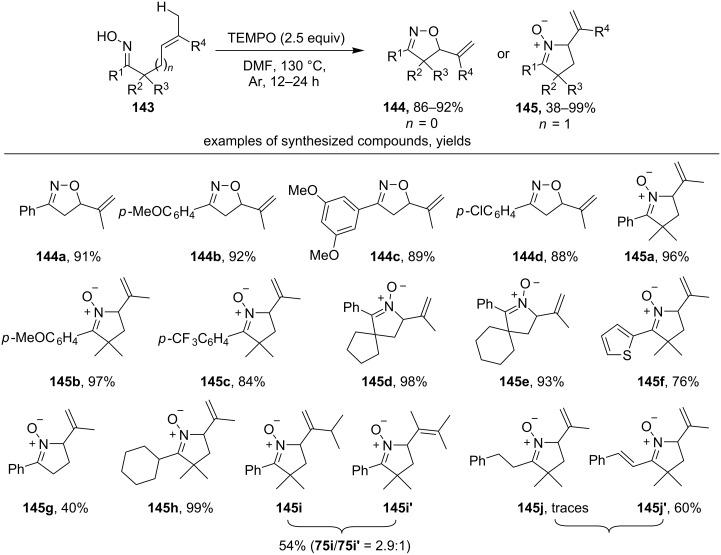
TEMPO-mediated oxidative cyclization of unsaturated oximes followed by elimination.

The reaction of TEMPO with β,γ- and γ,δ-unsaturated oximes **143** leads to substituted unsaturated isoxazolines **144** and cyclic nitrones **145**, respectively (Scheme 48) [137]. Presumably, the C-centered radical formed after 5-*exo-trig* cyclization of the oxime radical recombines with TEMPO. The resulting adduct undergoes β-elimination of TEMPOH with the formation of final unsaturated compounds. The intermediate coupling product of the C-centered radical and TEMPO was observed when the reaction was carried out at lower temperature (80 °C).

Isoxazolines **144** were synthesized from β,γ-unsaturated aryl oximes (R^1^ = Ar) with electron-donating (examples **144a–c**) and moderately electron-withdrawing substituents (example **144d**). The reaction of γ,δ-unsaturated aliphatic and aryl oximes with TEMPO yielded cyclic nitrones (products **145a–j**). The majority of the synthesized cyclic nitrones were disubstituted in α-position to the C=NO group (**145a–f,h–j**), the unsubstituted nitrone **145g** was synthesized in the lowest yield. In the case of **145j–145j’** the dehydrogenation of the side chain attached to C=NO fragment was observed.

**Scheme 49:**
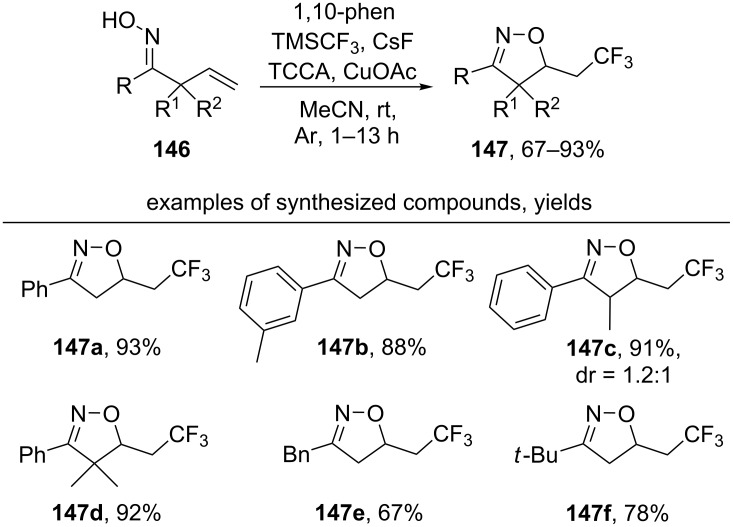
Oxidative cyclization of β,γ-unsaturated oximes with the introduction of a trifluoromethyl group.

Under the action of the TMSCF_3_/trichloroisocyanuric acid/TCCA/CuOAc/CsF system β,γ-unsaturated oximes **146** undergo oxidative cyclization to form substituted isoxazolines **147** with a trifluoromethyl group (Scheme 49) [138]. The authors note that the presence of 1,10-phenanthroline is necessary to stabilize the intermediate CuCF_3_.

Aromatic oximes with various electron-donating and electron-withdrawing substituents afford cyclization products in high yields (products **147a–d**). In addition to aryl oximes, benzyl and *tert*-butyl substituted oximes (products **147e**,**f**) were successfully used.

**Scheme 50:**
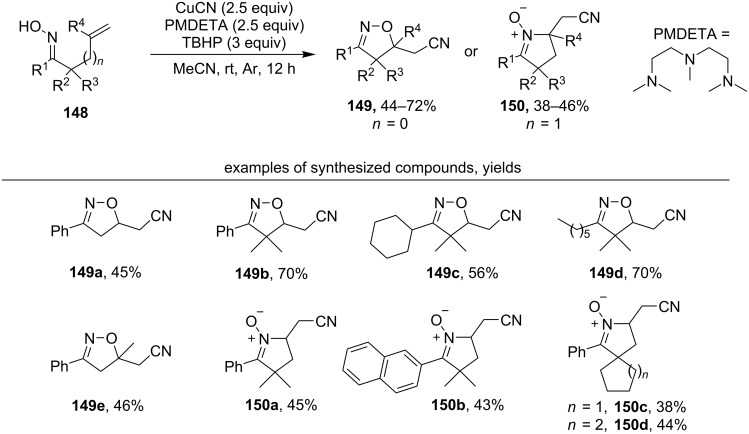
Oxidative cyclization of unsaturated oximes with the introduction of a nitrile group.

Oxidative cyclization of unsaturated oximes with the formation of isoxazolines or cyclic nitrones and the introduction of a nitrile group was achieved using the CuCN/*N*,*N*,*N*′,*N*′′,*N*′′-pentamethyldiethyltriamine (PMDETA)/TBHP system (Scheme 50) [139]. Other aliphatic amine ligands (*N*,*N*,*N*′,*N*′-tetramethylethylenediamine and 1,1,4,7,10,10-hexamethyltriethylenetetramine) showed moderate results and aromatic nitrogen-containing ligands (2,2’-bipyridine and phenanthroline) were even less efficient for the synthesis of target the isoxazolines.

Both aromatic (products **149a**,**b**,**e**) and aliphatic oximes (products **149c**,**d**) undergo this transformation with the formation of isoxazolines. Oximes with a disubstituted double bond also enter this reaction (product **149e**). The 5-*exo-trig* cyclization of γ,δ-unsaturated oximes under these conditions leads to substituted cyclic nitrones (products **150a–d**).

**Scheme 51:**
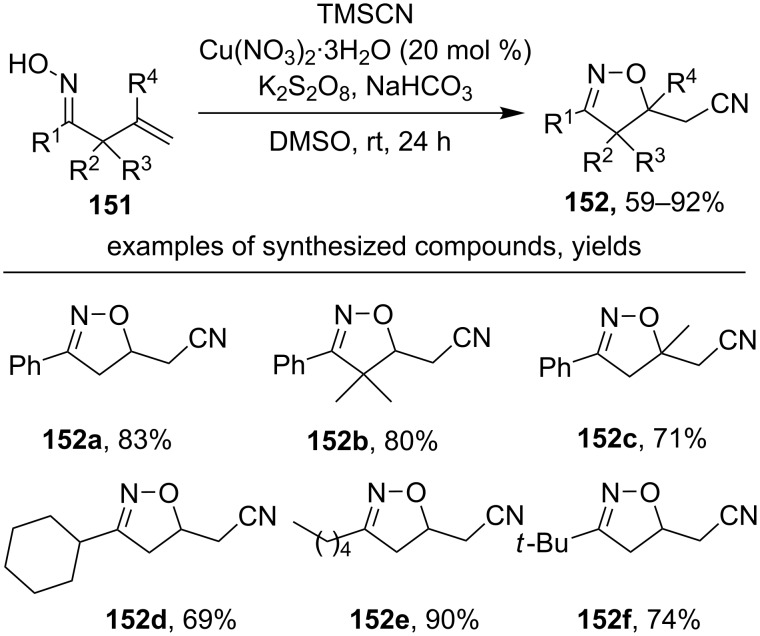
Oxidative cyclization of β,γ-unsaturated oximes to isoxazolines with the introduction of a nitrile group.

A similar cyanation reaction was realized using TMSCN as a cyanide source and the oxidative system Cu(NO_3_)_2_/K_2_S_2_O_8_ (Scheme 51) [140].

Both aromatic (products **152a–c**) and aliphatic (products **152d–f**) β,γ-unsaturated oximes undergo this transformation. Oximes substituted at the α-position (R^1^, R^2^ = Me), as well as oximes having a disubstituted double bond, also give cyclization products with good yields (products **152b,c**).

**Scheme 52:**
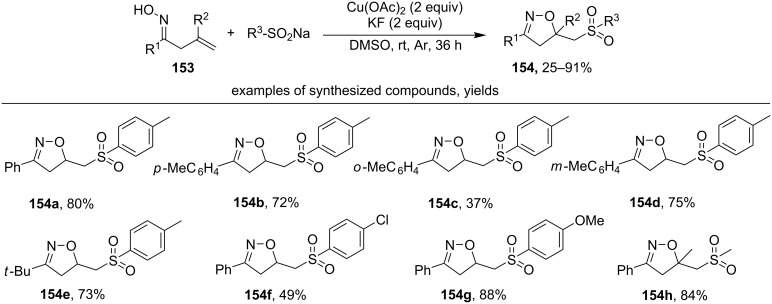
Oxidative cyclization of β,γ-unsaturated oximes to isoxazolines with the introduction of a sulfonyl group.

The interaction of β,γ-unsaturated oximes **153** with sodium sulfinates in the presence of copper(II) acetate leads to substituted isoxazolines **154** with the sulfonyl group (Scheme 52) [141].

Oximes, mainly aromatic ones, cyclize effectively (products **154a–d**,**f–h**). Examples **154b–d** demonstrate the effect of substituents in the phenyl ring of oxime (R^1^) on the product yield. When a substituent is present in the *ortho*-position of the benzene ring, the yield decreases compared to *para-* and *meta-*substituted substrates. In addition to sodium aryl sulfinates, sodium methane sulfinate was used for the synthesis of product **154h** in good yield. The presence of an electron-withdrawing group in the aromatic ring of sulfinate decreases the yield (product **154f** compared to product **154a**), and the presence of an electron-donating group increases the yield of isoxazoline (product **154g** compared to product **154a**).

**Scheme 53:**
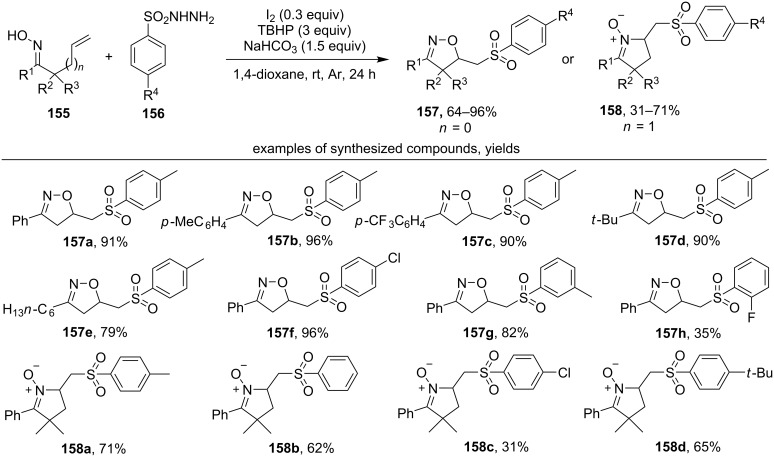
Oxidative cyclization of β,γ- and γ,δ-unsaturated oximes to isoxazolines with the introduction of a sulfonyl group.

Another approach to the synthesis of isoxazolines with a sulfonyl moiety is the reaction of unsaturated oximes **155** with sulfonyl hydrazides **156** under the action of TBHP and a catalytic amount of iodine (Scheme 53) [142].

Aromatic β,γ-unsaturated oximes containing both electron-donating and electron-withdrawing substituents in the phenyl ring give cyclization products in good yields (products **157a–c**). Aliphatic oximes also enter this reaction effectively (products **157d**,**e**). Under the same reaction conditions aromatic γ,δ-unsaturated oximes give sulfonyl-substituted cyclic nitrones (products **158a–d**).

**Scheme 54:**
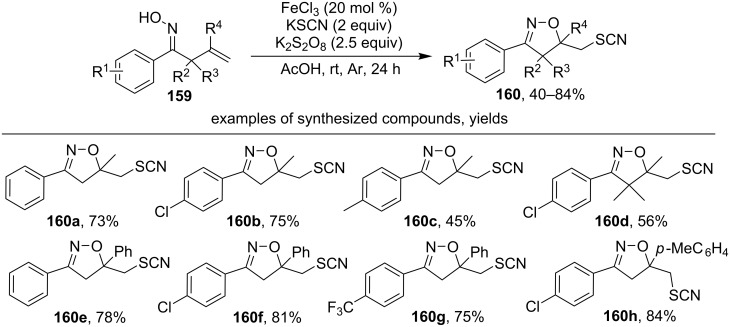
Oxidative cyclization of β,γ-unsaturated oximes to isoxazolines with the introduction of a thiocyanate group.

Another example of the oxidative cyclization of oximes with the formation of an isoxazoline ring and C–S bond is the reaction of aromatic β,γ-unsaturated oximes **159** with the FeCl_3_/KSCN/K_2_S_2_O_8_ system that affords thiocyanates **160** (Scheme 54) [143].

**Scheme 55:**
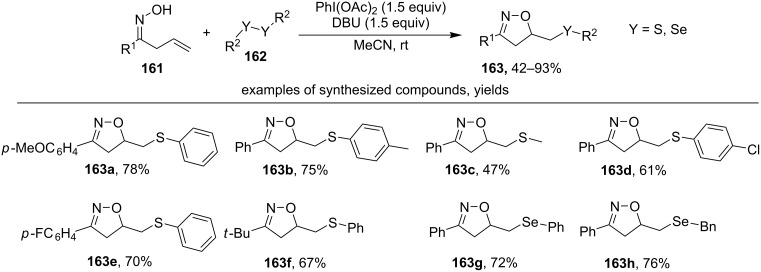
PhI(OAc)_2_-mediated oxidative cyclization of oximes with C–S and C–Se bond formation.

Under the action of PIDA (PhI(OAc)_2_), β,γ-unsaturated oximes **161** react with diselenides and disulfides **162** to form isoxazolines **163** (Scheme 55) [144].

Various disulfides of both aromatic (products **163a**,**b**,**d–f**) and aliphatic nature (product **163c**) were used. The presence of electron-donating substituents in the *para*-position of the phenyl ring of disulfide increases the yield of the isoxazoline (example **163b**), compared to electron-withdrawing substituents (example **163d**). Like disulfides, diselenides lead to the corresponding Se-containing isoxazolines **163g**,**h** in high yields. β,γ-Unsaturated tosyl hydrazones react with disulfides and diselenides analogously to oximes [144].

**Scheme 56:**
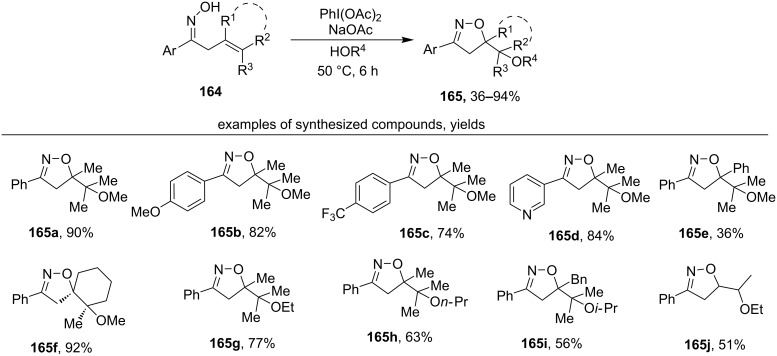
PhI(OAc)_2_-mediated oxidative cyclization of unsaturated oximes accompanied by alkoxylation.

Under the action of the PIDA/NaOAc/HOAlkyl system, β,γ-unsaturated aryl oximes **164** undergo oxidative cyclization with the formation of substituted isoxazolines **165** containing an ether group (Scheme 56) [145]. The alcohol acts both as a solvent and as a reagent in this transformation.

Most of the tested oximes contained a tetrasubstituted C=C double bond (examples **165a–i**) but the product of cyclization involving a disubstituted C=C double bond was also reported (example **165j)**. The ionic pathway was proposed for the formation of the products **165** as the most plausible but free radical pathway involving iminoxyl radicals was not ruled out [145]. In both considered reaction pathways the final stage was the formation of ether C–O bond by a nucleophilic attack of the intermediate carbocation by the alcohol.

**Scheme 57:**
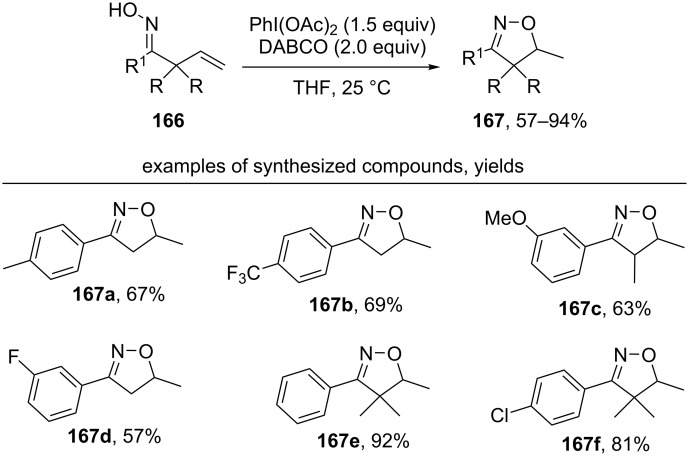
PhI(OAc)_2_-mediated cyclization of unsaturated oximes to methylisoxazolines.

Under the action of the related oxidative system PhI(OAc)_2_/DABCO in THF, cyclization of allyl-substituted oximes **166** without the introduction of a functional group at the terminal carbon atom was realized (Scheme 57) [146].

The authors proposed a mechanism in which a 5-*exo-trig* cyclization of the iminoxyl radical formed from oxime **166** under the action of the PhI(OAc)_2_/DABCO system produced the primary C-centered radical. Presumably, the final product **167** is formed via hydrogen atom transfer from THF to the intermediate C-centered radical [146]. This process is similar to the cobalt-catalyzed cyclization in the presence of 1,4-cyclohexadiene as hydrogen atom donor discussed above (Scheme 35) [125].

**Scheme 58:**
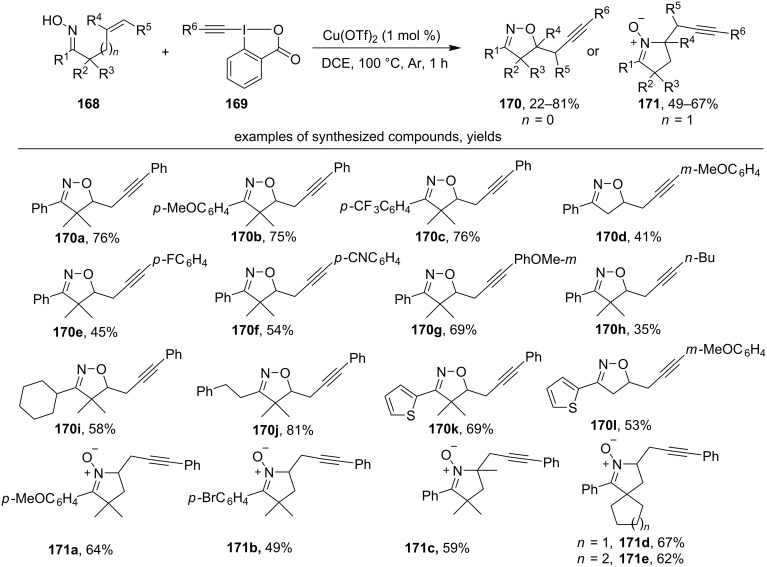
Oxidative cyclization-alkynylation of unsaturated oximes.

The reaction of unsaturated oximes **168** with ethynylbenziodoxolone (EBX) reagents **169** in the presence of copper(II) triflate leads to substituted isoxazolines **170** and cyclic nitrones **171** with an alkynyl group (Scheme 58) [147].

Various aromatic and some aliphatic oximes were tested as substrates (products **170a–l**). The yield of the reaction product is weakly dependent on the substituent in the benzene ring (examples **170a**,**b**,**c**). Oximes unsubstituted in α-position (R^2^ = R^3^ = H) also undergo this transformation (example **170d**,**l**). Both various aromatic substituents (products **170e–g**) and alkyl substituents (example **170h**) may be present in the EBX reagent at R^6^. Under the same reaction conditions, γ,δ-unsaturated oximes give substituted cyclic nitrones (products **171a–e**).

**Scheme 59:**
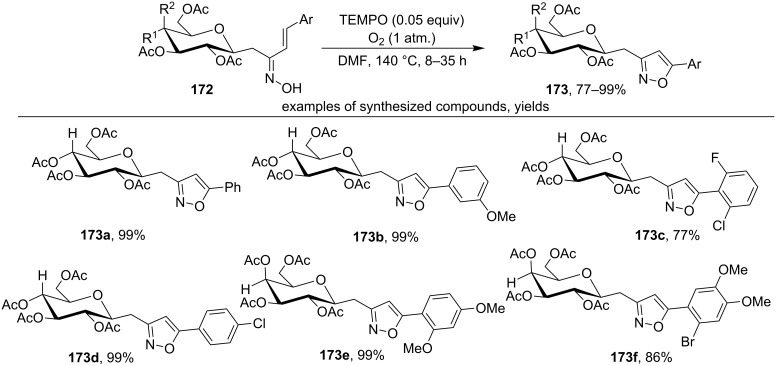
TEMPO-mediated oxidative cyclization of C-glycoside ketoximes to C-glycosylmethylisoxazoles.

The oxidative cyclization of C-glycoside ketoximes **172** was carried out under the action of catalytic amounts of TEMPO under oxygen (1 atm) with the formation of substituted isoxazoles **173** (Scheme 59) [148].

The proposed mechanism includes the oxidation of starting oxime to iminoxyl radical by TEMPO, 5-*endo-trig* cyclization and oxidative aromatization with the formation of the final isoxazole **173** [148].

**Scheme 60:**
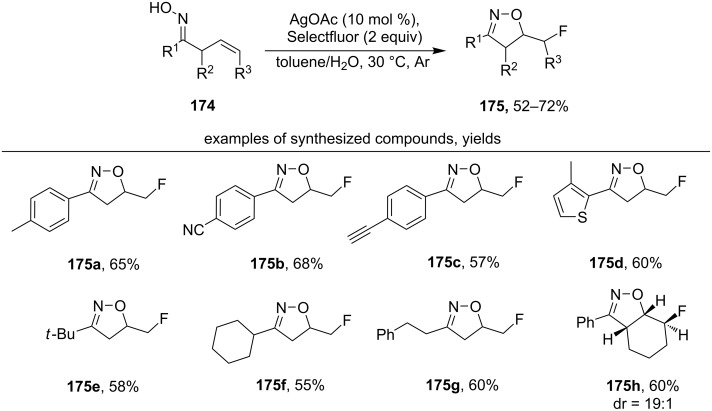
Silver-сatalyzed oxidative cyclization of β,γ-unsaturated oximes with formation of fluoroalkyl isoxazolines.

Under the action of the Selectfluor/AgOAc system, β,γ-unsaturated oximes **174** undergo oxidative cyclization with the formation of fluoroalkyl isoxazolines **175** (Scheme 60) [149].

The majority of products were fluoromethyl isoxazolines (examples **175a–g**). The exception was the product **175h**, which was synthesized from oxime with an endocyclic C=C double bond. The proposed mechanism includes the 5-*exo-trig* cyclization of the intermediate iminoxyl radical followed by fluorination of the formed C-centered radical. The 5-*exo-trig* radical cyclization was confirmed by a control experiment with the addition of TEMPO as a trapping reagent for the C-centered radical. The TEMPO-adduct was obtained in 75% yield [149].

**Scheme 61:**
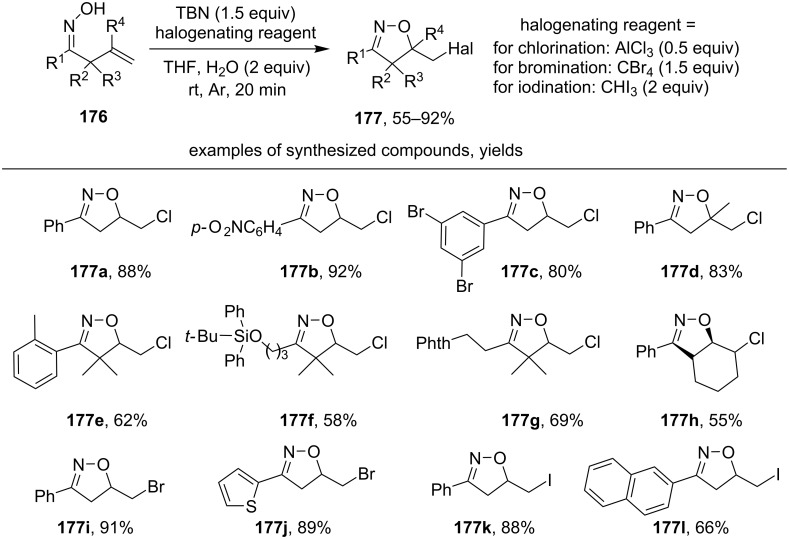
Oxidative cyclization of β,γ-unsaturated oximes with the formation of haloalkyl isoxazolines.

The formation of haloalkyl isoxazolines **177** was achieved upon the interaction of β,γ-unsaturated oximes **176** with *t*-BuONO and selected halogenating agents (Scheme 61) [150].

Both aromatic (products **177a–e**,**g**) and aliphatic oximes (products **177f**,**g**) successfully participate in this transformation. The yield of the reaction product weakly depends on the substituents in the aromatic ring of oxime (products **177a**,**b**,**c**). Oximes with a disubstituted double bond (R^4^ = Me, product **177d**), as well as α-unsubstituted or α-disubstituted oximes (R^2^ = R^3^ = H or Me) also produce isoxazolines with high yields (products **177a–d** and **177e–h**). Bromination and iodination of some substrates using CBr_4_ and CHI_3_ instead of AlCl_3_ were carried out (products **177i–l**). The radical mechanism including 5-*exo-trig* cyclization of the intermediate iminoxyl radical was supported by a radical trapping control experiment with TEMPO and a radical clock experiment with cyclopropane ring opening [150].

**Scheme 62:**
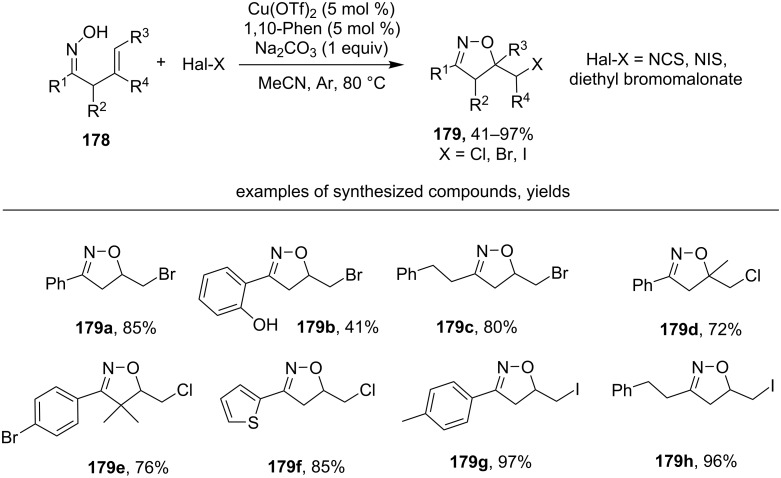
Cyclization of β,γ-unsaturated oximes into haloalkyl isoxazolines under the action of the halogenating reagent/Cu(OTf)_2_/phenantroline/Na_2_CO_3_ system.

A similar transformation of β,γ-unsaturated oximes **178** into haloalkylisoxazolines **179** was realized employing the catalytic system halogenating reagent/Cu(OTf)_2_/phenantroline/Na_2_CO_3_ (Scheme 62) [151]. The authors proposed an analogous mechanism involving 5-*exo-trig* cyclization of intermediate iminoxyl radical.

**Scheme 63:**
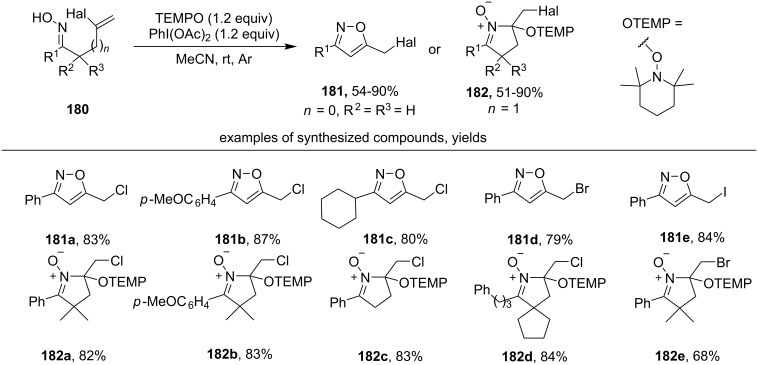
Synthesis of haloalkyl isoxazoles and cyclic nitrones via oxidative cyclization and 1,2-halogen shift.

Under the action of the PhI(OAc)_2_/TEMPO system on halogen-substituted unsaturated oximes **180**, halomethyl isoxazoles **181** and cyclic nitrones **182** are formed (Scheme 63) [152]. The authors note that the reaction proceeds through the 5-*exo-trig* cyclization of iminoxyl radical followed by a 1,2-halogen radical shift.

Chloro-, bromo-, and iodosubstituted β,γ-unsaturated oximes enter the reaction effectively (products **181a–e**). When γ,δ-unsaturated oximes were used instead of β,γ-unsaturated oximes, the formation of cyclic nitrones with a TEMPO fragment (products **182a–e**) was observed. Chloro- and bromo-substituted γ,δ-unsaturated oximes were applicable for this transformation, but iodo-substituted γ,δ-oximes gave target products **182** only in trace amounts.

**Scheme 64:**
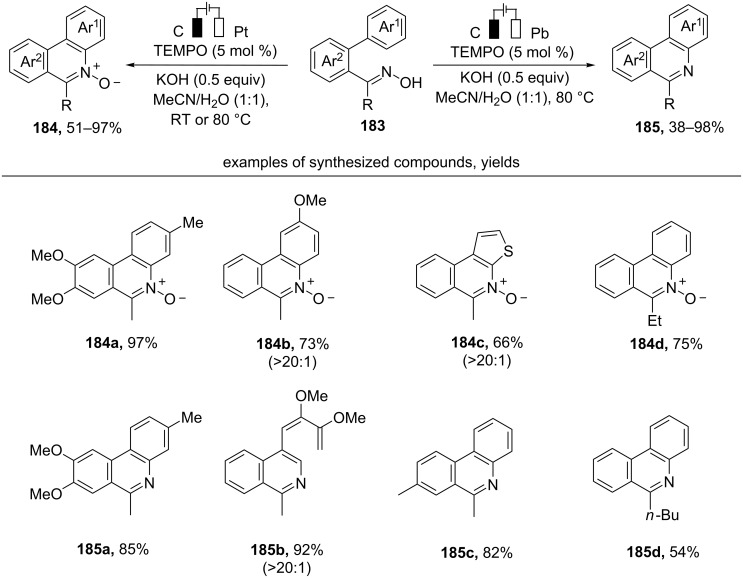
Electrochemical oxidative cyclization of diaryl oximes.

The TEMPO-mediated electrochemical cyclization of biaryl oximes **183** leads to *N*-heteroaromatic *N*-oxides **184** or *N*-heteroaromatic compounds **185** depending on a cathode material (Scheme 64) [153]. Reactions were performed in an undivided cell under constant current conditions. Reticulated vitreous carbon (RVC) electrode was used as an anode.

The proposed mechanism includes the formation of an iminoxyl radical followed by its cyclization onto the phenyl ring and oxidative rearomatization to N-heteroaromatic *N*-oxide **184**. In the case of Pt-cathode it is the final product, and in the case of Pb-cathode it is reduced to the N-heteroaromatic compound **185** [153].

**Scheme 65:**
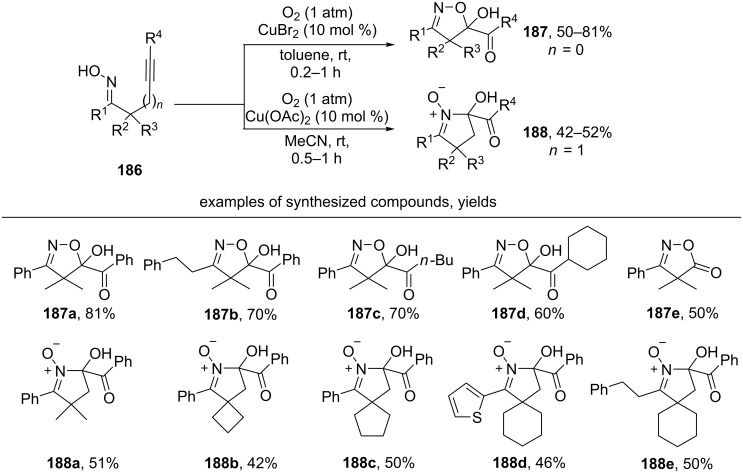
Copper-сatalyzed cyclization and dioxygenation oximes containing a triple C≡C bond.

The rare example of the intramolecular cyclization of iminoxyl radicals involving a triple bond is shown in Scheme 65 [154]. In the presence of catalytic amounts of copper(II) salt and molecular oxygen (1 atm) 5-*exo-dig* cyclization of unsaturated oximes **186** occurs.

Various β,γ-unsaturated oximes **186** react with the formation of substituted isoxazoline α-ketols **187a–d**. Using an oxime with a terminal triple bond, isoxazolone **187e** was obtained. Under analogous conditions γ,δ-unsaturated oximes afford cyclic nitrones **188a–e** in moderate yields.

**Scheme 66:**
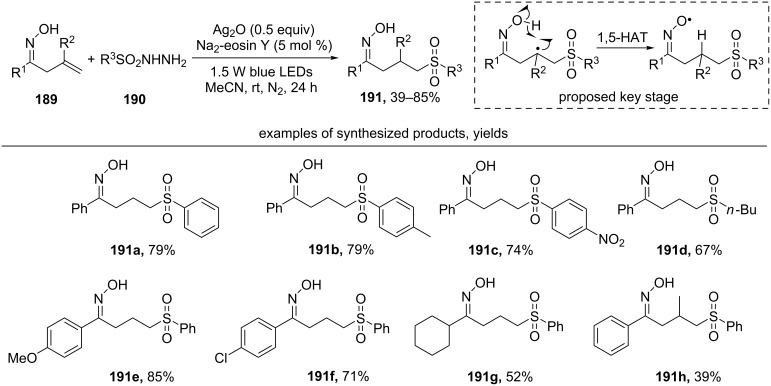
Photoredox-catalyzed sulfonylation of β,γ-unsaturated oximes by sulfonyl hydrazides.

Under irradiation of blue LED (450-455 nm) of the mixture of oximes **189**, sulfonyl hydrazides **190**, silver(I) oxide and disodium salt of eosin Y in an inert atmosphere sulfones **191** are formed [155] (Scheme 66). In this case, hydrosulfonylation of the C=C double bond takes place instead of typical cyclization of β,γ-unsaturated oximes **189** to isoxazolines.

Both aromatic (examples **191a–c, e–h**) and aliphatic (example **191d**) sulfonyl hydrazides undergo this reaction successfully. Good yields were obtained with both electron-donating (example **191b**) and electron-withdrawing substituents (example **191c**) in the aromatic ring of sulfonyl hydrazide. The introduction of a methyl group in the β-position relative to the oxime group leads to a decrease in yield (example **191h**).

The authors proposed a radical mechanism in which the addition of the sulfonyl radical to the double C=C bond, is followed by 1,5-HAT from the oxime hydroxy group to the carbon radical. An experiment with a deuterium label on the hydroxy group of oxime confirmed this hypothesis: the deuterium was found at the β-carbon atom of the product [155].

**Scheme 67:**
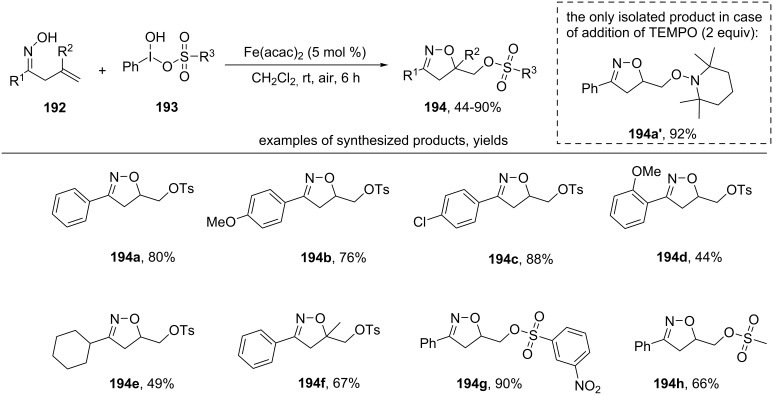
Oxidative cyclization of β,γ-unsaturated oximes with introduction of sulfonate group.

The combination of Koser’s reagent **193** and Fe(acac)_2_ as catalyst was used for the synthesis of sulfonates **194** from unsaturated oximes **192** [156] (Scheme 67).

In the presence of a substituent in the *ortho*-position of the benzene ring, the yield of the product decreases remarkably, probably due to steric hindrances during the reaction (example **194d**). Aliphatic oximes (example **194e**) and oximes with a disubstituted C=C bond (example **194f**) undergo this transformation with moderate yields. Free-radical mechanism involving the cyclization of the iminoxyl radical was confirmed by trapping of a C-centered radical, TEMPO-adduct **194a’** was isolated in 92% yield instead of **194a** when TEMPO was added to the reaction mixture. The authors also showed that the presence of water in the system can lead to the formation of alcohol instead of sulfonate [156].

**Scheme 68:**
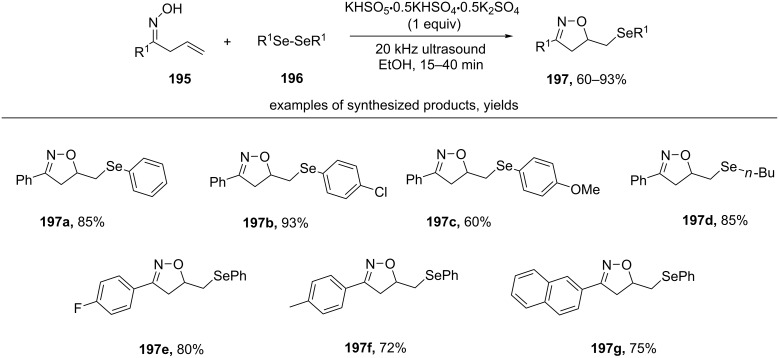
Ultrasound-promoted oxidative cyclization of β,γ-unsaturated oximes.

Under ultrasound irradiation β,γ-unsaturated oximes **195** react with KHSO_5_·0.5KHSO_4_·0.5K_2_SO_4_ and diselenides **196** to form isoxazolines **197** [157] (Scheme 68)

The introduction of electron-withdrawing substituents (example **197b**) into the benzene ring of diselenide leads to an increase in yield compared to the case of electron-donating substituents (example **197c**). Dialkyl diselenides also undergo this transformation (example **197d**). Similar yields of the target product **197a** were obtained when rthe eaction mixture was heated by microwave irradiation or an oil bath instead of sonication. However, control experiments allowed to suggest that the reaction can proceed by both radical and ionic mechanisms. The authors proposed that under ultrasonic stimulation the mechanism is predominantly radical, while under microwave conditions the ionic mechanism becomes significant [157].

## Conclusion

An unprecedented renaissance in the chemistry of oxime radicals has been observed during the last years. Over the past decade, a diverse pattern of methods for oxidative cyclization involving oxime radicals was developed. 5-*Exo-trig* cyclization of oxime radicals generated from unsaturated oximes can now be considered as a robust and general approach to the synthesis of functionalized isoxazolines, isoxazoles, and cyclic nitrones. Moreover, iminoxyl radicals were demonstrated to be promising intermediates for cyclizations involving C(sp^3^)–H bonds, C≡C triple bonds and aromatic π-systems.

The majority of iminoxyl-radical-mediated synthetic transformations are intramolecular reactions, presumably due to the lack of stability of the oxime radicals. Nevertheless, examples of intermolecular cross-dehydrogenative C–O couplings employing oxime radicals as the key intermediates emerged recently.

The areas of the future development of the chemistry of iminoxyl radicals include: (1) the development of selective intermolecular reactions with oxime radicals; (2) the development of new oxidative systems for the generation of oxime radicals, including electrochemical and photochemical approaches; (3) the search for new stable oxime radicals which can be used as reagents for organic synthesis and other applications.

## References

[1] Tretyakov E V, Ovcharenko V I (2009). Russ Chem Rev.

[2] Muench S, Wild A, Friebe C, Häupler B, Janoschka T, Schubert U S (2016). Chem Rev.

[3] Nishide H, Iwasa S, Pu Y-J, Suga T, Nakahara K, Satoh M (2004). Electrochim Acta.

[4] Hansen K-A, Nerkar J, Thomas K, Bottle S E, O’Mullane A P, Talbot P C, Blinco J P (2018). ACS Appl Mater Interfaces.

[5] Gigmes D (2015). Nitroxide Mediated Polymerization: From Fundamentals to Applications in Materials Science.

[6] Audran G, Bagryanskaya E, Edeleva M, Marque S R A, Parkhomenko D, Tretyakov E, Zhivetyeva S (2018). Aust J Chem.

[7] Haugland M M, Lovett J E, Anderson E A (2018). Chem Soc Rev.

[8] Kim Y, Wang Y, Chen H-Y, Hilty C, Ghose R (2018). In-Vitro Dissolution Dynamic Nuclear Polarization for Sensitivity Enhancement of NMR with Biological Molecules. Protein NMR.

[9] Bagryanskaya E G, Marque S R A (2014). Chem Rev.

[10] Cao Q, Dornan L M, Rogan L, Hughes N L, Muldoon M J (2014). Chem Commun.

[11] Ciriminna R, Ghahremani M, Karimi B, Pagliaro M (2017). ChemistryOpen.

[12] Recupero F, Punta C (2007). Chem Rev.

[13] Galli C, Gentili P, Lanzalunga O (2008). Angew Chem, Int Ed.

[14] Coseri S (2009). Catal Rev: Sci Eng.

[15] Melone L, Punta C (2013). Beilstein J Org Chem.

[16] Wertz S, Studer A (2013). Green Chem.

[17] Chen K, Zhang P, Wang Y, Li H (2014). Green Chem.

[18] Chen K, Xie H (2017). Chin J Catal.

[19] Lan X-W, Wang N-X, Xing Y (2017). Eur J Org Chem.

[20] Bag R, De P B, Pradhan S, Punniyamurthy T (2017). Eur J Org Chem.

[21] Giglio B C, Alexanian E J (2014). Org Lett.

[22] Quinn R K, Schmidt V A, Alexanian E J (2013). Chem Sci.

[23] Schmidt V A, Alexanian E J (2012). Chem Sci.

[24] Giglio B C, Schmidt V A, Alexanian E J (2011). J Am Chem Soc.

[25] Schmidt V A, Alexanian E J (2011). J Am Chem Soc.

[26] Schmidt V A, Alexanian E J (2010). Angew Chem, Int Ed.

[27] Punta C, Rector C L, Porter N A (2005). Chem Res Toxicol.

[28] Jousserandot A, Boucher J-L, Henry Y, Niklaus B, Clement B, Mansuy D (1998). Biochemistry.

[29] Sturgeon B E, Glover R E, Chen Y-R, Burka L T, Mason R P (2001). J Biol Chem.

[30] Papavasileiou K D, Tzima T D, Sanakis Y, Melissas V S (2007). ChemPhysChem.

[31] Sanakis Y, Goussias C, Mason R P, Petrouleas V (1997). Biochemistry.

[32] Gunther M R, Hsi L C, Curtis J F, Gierse J K, Marnett L J, Eling T E, Mason R P (1997). J Biol Chem.

[33] Gunther M (2002). Toxicology.

[34] Okazaki R, Inagaki Y (1978). J Synth Org Chem, Jpn.

[35] Ingold K U, Hicks R G (2010). The Only Stable Organic Sigma Radicals: Di‐tert ‐Alkyliminoxyls. Stable Radicals.

[36] Ingold K U, Fischer H (1983). Iminoxyl radicals, RR’C=NO. Radicals Centered on N, S, P and Other Heteroatoms. Nitroxyls.

[37] Fischer H, Hellwege K-H (1979). Organic N-Centered Radicals and Nitroxide Radicals.

[38] Thomas J R (1964). J Am Chem Soc.

[39] Stone T J, Waters W A (1962). Proc Chem Soc, London.

[40] Krylov I B, Kompanets M O, Novikova K V, Opeida I O, Kushch O V, Shelimov B N, Nikishin G I, Levitsky D O, Terent’ev A O (2016). J Phys Chem A.

[41] Amorati R, Lucarini M, Mugnaini V, Pedulli G F, Minisci F, Recupero F, Fontana F, Astolfi P, Greci L (2003). J Org Chem.

[42] Flesia E, Surzur J-M, Tordo P (1978). Org Magn Reson.

[43] Rozantsev E G, Ulrich H (1970). Free Nitroxyl Radicals.

[44] Krylov I B, Paveliev S A, Shelimov B N, Lokshin B V, Garbuzova I A, Tafeenko V A, Chernyshev V V, Budnikov A S, Nikishin G I, Terent'ev A O (2017). Org Chem Front.

[45] Mendenhall G D, Ingold K U (1973). J Am Chem Soc.

[46] Krylov I B, Terent'ev A O, Timofeev V P, Shelimov B N, Novikov R A, Merkulova V M, Nikishin G I (2014). Adv Synth Catal.

[47] Gilbert B C, Norman R O C, Price D C (1964). Proc Chem Soc, London.

[48] Lemaire H, Rassat A (1964). Tetrahedron Lett.

[49] Gilbert B C, Norman R O C (1966). J Chem Soc B.

[50] Fox W M, Symons M C R (1966). J Chem Soc A.

[51] Butler R N, Scott F L, O'Mahony T A F (1973). Chem Rev.

[52] Koch R, Wollweber H-J, Müller-Starke H, Wentrup C (2015). Eur J Org Chem.

[53] Brokenshire J L, Roberts J R, Ingold K U (1972). J Am Chem Soc.

[54] Siatecki Z, Chmielewski P J, Jezierski A (1992). Magn Reson Chem.

[55] Lagercrantz C, Ekström M, Spassov S, Hörnfeldt A-B, Lönnberg H, Berg J-E, Bartók M, Pelczer I, Dombi G (1988). Acta Chem Scand, Ser B.

[56] Lin T-s, Mastin S H, Ohkaku N (1973). J Am Chem Soc.

[57] Petrosyan V A, Niyazymbetov M E, Ul’yanova é V (1990). Russ Chem Bull.

[58] Fukunishi K, Kitada K, Naito I (1991). Synthesis.

[59] Brokenshire J L, Mendenhall G D, Ingold K U (1971). J Am Chem Soc.

[60] Eisenhauer B M, Wang M, Brown R E, Labaziewicz H, Ngo M, Kettinger K W, Mendenhall G D (1997). J Phys Org Chem.

[61] Eisenhauer B M, Wang M, Labaziewicz H, Ngo M, Mendenhall G D (1997). J Org Chem.

[62] Lindsay D, Horswill E C, Davidson D W, Ingold K U (1974). Can J Chem.

[63] Lagercrantz C, Torssell K (1967). Ark Kemi.

[64] Hoffman S, Jezierski A, Jezowska-Trzebiatowska B (1986). Bull Pol Acad Sci, Chem.

[65] Zeilstra J J, Engberts J B F N (1973). Tetrahedron.

[66] Symons M C R (1965). J Chem Soc.

[67] Chong S-S, Fu Y, Liu L, Guo Q-X (2007). J Phys Chem A.

[68] Lucarini M, Pedulli G F, Alberti A (1994). J Org Chem.

[69] Alberti A, Barbaro G, Battaglia A, Guerra M, Bernardi F, Dondoni A, Pedulli G F (1981). J Org Chem.

[70] Pratt D A, Blake J A, Mulder P, Walton J C, Korth H-G, Ingold K U (2004). J Am Chem Soc.

[71] Dao R, Wang X, Chen K, Zhao C, Yao J, Li H (2017). Phys Chem Chem Phys.

[72] Mahoney L R, Mendenhall G D, Ingold K U (1973). J Am Chem Soc.

[73] Mickel M, Kim H-C, Hampp N (2003). Green Chem.

[74] Chhabra M, Mishra S, Sreekrishnan T R (2015). Biochem Eng J.

[75] Morozova O V, Shumakovich G P, Shleev S V, Yaropolov Y I (2007). Appl Biochem Microbiol.

[76] Galli C, Gentili P (2004). J Phys Org Chem.

[77] Coseri S, Mendenhall G D, Ingold K U (2005). J Org Chem.

[78] Ngo M, Larson K R, Mendenhall G D (1986). J Org Chem.

[79] Mahoney L R, DaRooge M A (1970). J Am Chem Soc.

[80] Mulder P, Saastad O W, Griller D (1988). J Am Chem Soc.

[81] Jonsson M, Lind J, Eriksen T E, Merényi G (1993). J Chem Soc, Perkin Trans 2.

[82] Wayner D D M, Lusztyk E, Ingold K U, Mulder P (1996). J Org Chem.

[83] Pratt D A, de Heer M I, Mulder P, Ingold K U (2001). J Am Chem Soc.

[84] Pratt D A, DiLabio G A, Mulder P, Ingold K U (2004). Acc Chem Res.

[85] Cornejo J J, Larson K D, Mendenhall G D (1985). J Org Chem.

[86] Schenk C, De Boer T J (1979). Tetrahedron.

[87] Krylov I B, Paveliev S A, Shumakova N S, Syroeshkin M A, Shelimov B N, Nikishin G I, Terent'ev A O (2018). RSC Adv.

[88] Bityukov O V, Vil' V A, Sazonov G K, Kirillov A S, Lukashin N V, Nikishin G I, Terent'ev A O (2019). Tetrahedron Lett.

[89] Terent'ev A O, Zdvizhkov A T, Levitsky D O, Fleury F, Pototskiy R A, Kulakova A N, Nikishin G I (2015). Tetrahedron.

[90] Terent'ev A O, Sharipov M Y, Krylov I B, Gaidarenko D V, Nikishin G I (2015). Org Biomol Chem.

[91] Terent´ev A O, Vil´ V A, Bityukov O V, Nikishin G I (2014). Russ Chem Bull.

[92] Terent’ev A, Borisov D, Semenov V, Chernyshev V, Dembitsky V, Nikishin G (2011). Synthesis.

[93] Terent’ev A O, Borisov D A, Yaremenko I A, Chernyshev V V, Nikishin G I (2010). J Org Chem.

[94] Krylov I B, Terent’ev A O (2015). Russ J Org Chem.

[95] Chen Z-Y, Liang H-J, Chen R-X, Chen L, Tang X-Z, Yan M, Zhang X-J (2019). Adv Synth Catal.

[96] Han Z, Shen S, Zheng F, Hu H, Zhang J, Zhu S (2019). Tetrahedron Lett.

[97] Lopes E F, Penteado F, Thurow S, Pinz M, Reis A S, Wilhelm E A, Luchese C, Barcellos T, Dalberto B, Alves D (2019). J Org Chem.

[98] Dong K-Y, Qin H-T, Bao X-X, Liu F, Zhu C (2014). Org Lett.

[99] Tripathi C B, Mukherjee S (2013). Angew Chem, Int Ed.

[100] Triandafillidi I, Kokotos C G (2017). Org Lett.

[101] Praveen C, Kalyanasundaram A, Perumal P (2010). Synlett.

[102] He Y-T, Li L-H, Yang Y-F, Wang Y-Q, Luo J-Y, Liu X-Y, Liang Y-M (2013). Chem Commun.

[103] Dong K-Y, Qin H-T, Liu F, Zhu C (2015). Eur J Org Chem.

[104] Sun Y, Abdukader A, Zhang H, Yang W, Liu C (2017). RSC Adv.

[105] Li X-T, Gu Q-S, Dong X-Y, Meng X, Liu X-Y (2018). Angew Chem, Int Ed.

[106] Jiang D, Peng J, Chen Y (2008). Org Lett.

[107] Zhu M-K, Zhao J-F, Loh T-P (2010). J Am Chem Soc.

[108] Li X-T, Lv L, Gu Q-S, Liu X-Y (2018). Tetrahedron.

[109] Wei Q, Chen J-R, Hu X-Q, Yang X-C, Lu B, Xiao W-J (2015). Org Lett.

[110] Zhu M, Fun W, Guo W, Tian Y, Wang Z, Xu C, Ji B (2019). Eur J Org Chem.

[111] Zhang Y, Ji M (2019). Eur J Org Chem.

[112] Forrester A R, Thomson R H, Woo S-O (1975). J Chem Soc, Perkin Trans 1.

[113] Kong W, Guo Q, Xu Z, Wang G, Jiang X, Wang R (2015). Org Lett.

[114] Zhu X, Wang Y-F, Ren W, Zhang F-L, Chiba S (2013). Org Lett.

[115] Shotter R G, Sesardić D, Wright P H (1975). Tetrahedron.

[116] Shi D, Qin H-T, Zhu C, Liu F (2015). Eur J Org Chem.

[117] Zhang F-L, Wang Y-F, Chiba S (2013). Org Biomol Chem.

[118] Wang Y-F, Zhang F-L, Chiba S (2013). Org Lett.

[119] Lemercier B C, Pierce J G (2015). Org Lett.

[120] Parker P D, Pierce J G (2016). Synthesis.

[121] Liu Y-Y, Yang X-H, Yang J, Song R-J, Li J-H (2014). Chem Commun.

[122] Soni V K, Kim J, Cho E J (2018). Adv Synth Catal.

[123] Atmaram S, Forrester A R, Gill M, Napier R J, Thomson R H, Tezuka T (1982). Acta Chem Scand, Ser B.

[124] Han B, Yang X-L, Fang R, Yu W, Wang C, Duan X-Y, Liu S (2012). Angew Chem, Int Ed.

[125] Li W, Jia P, Han B, Li D, Yu W (2013). Tetrahedron.

[126] Yamamoto D, Oguro T, Tashiro Y, Soga M, Miyashita K, Aso Y, Makino K (2016). Eur J Org Chem.

[127] Hu X-Q, Chen J, Chen J-R, Yan D-M, Xiao W-J (2016). Chem – Eur J.

[128] Yang X-L, Chen F, Zhou N-N, Yu W, Han B (2014). Org Lett.

[129] Xu Y, Chen H, Li W, Xie Q, Yu L, Shao L (2018). Org Biomol Chem.

[130] Peng X-X, Deng Y-J, Yang X-L, Zhang L, Yu W, Han B (2014). Org Lett.

[131] Zhang X-W, Xiao Z-F, Zhuang Y-J, Wang M-M, Kang Y-B (2016). Adv Synth Catal.

[132] Wang D-J, Chen B-Y, Wang Y-Q, Zhang X-W (2018). Eur J Org Chem.

[133] Zhu L, Wang G, Guo Q, Xu Z, Zhang D, Wang R (2014). Org Lett.

[134] Zhu L, Yu H, Xu Z, Jiang X, Lin L, Wang R (2014). Org Lett.

[135] Yang X-L, Long Y, Chen F, Han B (2016). Org Chem Front.

[136] Liu R-H, Wei D, Han B, Yu W (2016). ACS Catal.

[137] Chen F, Yang X-L, Wu Z-W, Han B (2016). J Org Chem.

[138] Zhang W, Su Y, Wang K-H, Wu L, Chang B, Shi Y, Huang D, Hu Y (2017). Org Lett.

[139] Chen F, Zhu F-F, Zhang M, Liu R-H, Yu W, Han B (2017). Org Lett.

[140] Meng F, Zhang H, Guo K, Dong J, Lu A-M, Zhu Y (2017). J Org Chem.

[141] Wang L-J, Chen M, Qi L, Xu Z, Li W (2017). Chem Commun.

[142] Xu Z-Q, Zheng L-C, Li L, Duan L, Li Y-M (2019). Org Biomol Chem.

[143] Ji F, Fan Y, Yang R, Yang Y, Yu D, Wang M, Li Z (2017). Asian J Org Chem.

[144] Yu J-M, Cai C (2018). Org Biomol Chem.

[145] Ye C, Kou X, Yang G, Shen J, Zhang W (2019). Tetrahedron Lett.

[146] Hu X-Q, Feng G, Chen J-R, Yan D-M, Zhao Q-Q, Wei Q, Xiao W-J (2015). Org Biomol Chem.

[147] Han W-J, Wang Y-R, Zhang J-W, Chen F, Zhou B, Han B (2018). Org Lett.

[148] Llantén H, Barata-Vallejo S, Postigo A, Colinas P A (2017). Tetrahedron Lett.

[149] Liu Y-Y, Yang J, Song R-J, Li J-H (2014). Adv Synth Catal.

[150] Zhang X-W, Xiao Z-F, Wang M-M, Zhuang Y-J, Kang Y-B (2016). Org Biomol Chem.

[151] Li X, Ding Y, Qian L, Gao Y, Wang X, Yan X, Xu X (2019). J Org Chem.

[152] Chen H-L, Wei D, Zhang J-W, Li C-L, Yu W, Han B (2018). Org Lett.

[153] Zhao H-B, Xu P, Song J, Xu H-C (2018). Angew Chem, Int Ed.

[154] Peng X-X, Wei D, Han W-J, Chen F, Yu W, Han B (2017). ACS Catal.

[155] Yang S, Wang L, Wang L, Li H (2020). J Org Chem.

[156] Yang S, Li H, Li P, Yang J, Wang L (2020). Org Biomol Chem.

[157] Araujo D R, Lima Y R, Barcellos A M, Silva M S, Jacob R G, Lenardão E J, Bagnoli L, Santi C, Perin G (2020). Eur J Org Chem.

